# Relational influences on help-seeking for mental health and substance use problems among people experiencing social marginalisation: a scoping review

**DOI:** 10.1136/bmjopen-2024-090349

**Published:** 2025-06-04

**Authors:** Catriona Connell, David Griffiths, Richard Kjellgren, Jessica Greenhalgh

**Affiliations:** 1 University of Stirling, Stirling, UK

**Keywords:** Substance misuse, MENTAL HEALTH, Decision Making

## Abstract

**Abstract:**

**Objectives:**

Understand the relational influences on help-seeking for mental health and substance use problems among people experiencing social marginalisation, with a focus on research applying social capital theory and social network analysis methods.

**Design:**

Scoping review.

**Data sources:**

EMBASE, Web of Science, Criminal Justice Abstracts and SocINDEX were searched up to June 2023, and Web of Science email alerts were used to capture any further publications up to June 2024.

**Eligibility criteria:**

English-language, peer-reviewed publications that (1) focused on/discussed help-seeking for mental health or substance use problems; (2) included adults experiencing social marginalisation beyond sociodemographic factors; and (3) applied social capital theory or social network analysis methods.

**Data extraction and synthesis:**

We extracted and charted data pertinent to review objectives and narratively synthesised results.

**Results:**

Twenty-seven papers were included. Most (n=19) focused on the experiences of people who used drugs. Five specifically focused on help-seeking, four of which applied quantitative social network analysis, one was framed by network theories of social capital and one referred to social capital in interpreting findings. The remaining 22 papers discussed help-seeking while focused on different phenomena. Seven of these framed their approach with social capital, but none explicitly applied social capital to help-seeking. Eight papers used social network analysis, with four focused on help-seeking and seven using personal networks. Social/relational influences identified included: fear of losing social capital, the risks of high bonding capital, service providers as social capital, selective help-seeking, trust and network culture. Social capital, interconnected with the tight-knit bonds within marginalised groups, could deter help-seeking. Knowledge and attitudes towards help and help-seeking, shaped by past experiences and network cultures, influenced help-seeking and contributed to a cautious and selective approach.

**Conclusion:**

Theoretical elaboration and empirical research are required to better appreciate the relational influences on help-seeking for mental health and substance use problems among people experiencing social marginalisation. Social capital may provide a useful theoretical approach. While social network analysis methods have been applied, they are under-utilised.

**STRENGTHS AND LIMITATIONS OF THIS STUDY**

- Applied rigorous scoping review methods and tested a quality appraisal tool on studies using social network analysis.
- Identified important relational influences that encourage or deter help-seeking among people experiencing social marginalisation, with implications for intervention planning.
- Papers may have been overlooked due to variation in terminology and concepts related to help-seeking.
- Studies of other types of social marginalisation, or applying other social theories or research methods, were not included.

## Introduction

 People marginalised by circumstances such as homelessness or justice system involvement have higher levels of mental health (MH) and substance use (SU) problems than the general population.^1 2^ While evidence-based treatments for MH and SU problems are available, these are not accessed equitably across populations,^3^ suggesting that certain groups experience greater barriers to access than others. When MH and/or SU are not addressed with effective treatment and support, there is an increased risk a person will reach a point of crisis that brings them to the attention of emergency health and other services. Indeed, those experiencing homelessness or with justice involvement in their lives are disproportionately represented in attendances at emergency services.^4 5^ MH and SU are the primary reasons for attendance among these groups and a driver of frequent attendance.^5 6^ This high use of emergency care indicates that people experiencing social marginalisation may face barriers to accessing early and preventative MH and SU services.

Accessing healthcare depends on interrelated demand and supply elements, spanning systemic, social, cultural and personal factors. Levesque *et al*^7^ describe five distinct but interrelated components required for access: identifying healthcare needs; seeking healthcare services; reaching healthcare services; using services; and being offered appropriate services. Barriers and facilitators (which may be personal, social, cultural, structural or organisational) act on different components to affect access, and their impact will differ between people with different characteristics, in different contexts and when accessing different types of healthcare. For example, among people experiencing justice involvement, barriers to service access include being unable to register with a general practitioner, stigma within society and services, cost (where health and social care is not funded by general taxation), service fragmentation and frequent change in providers, service designs that do not account for multi-morbidity, and waiting lists.^8^,^11^

Help-seeking is a fundamental component of accessing treatment and support.^7^ It involves responding to perceived needs by actively seeking assistance, support or guidance from various sources, including informal networks, community resources and professional services. Research into help-seeking for MH or SU often uses healthcare utilisation rates as a measure of help-seeking, which does not distinguish between the act of seeking help and successfully accessing treatment.^12 13^ We regard help-seeking to be the steps individuals take to identify and reach out for help, regardless of whether they ultimately successfully access that help. This interpretation can include individuals not currently accessing any help despite trying to, while excluding those court-mandated to receive services they feel they do not need.

There may be specific social and relational dynamics related to help-seeking for MH and SU compared with help-seeking in general, as MH and SU carry high levels of social stigma. There are differences in MH and SU stigma depending on the nature and severity of someone’s presentation (eg, some MH conditions are less stigmatised than others, and there are different attitudes and legal approaches towards illicit drugs and alcohol). Additionally, stigma may operate differently to impact help-seeking among people who experience different forms of social marginalisation,^14^ for instance, based on sociodemographic factors such as gender, ethnicity, social class and their intersections. However, evidence gaps have been identified in understanding help-seeking among people who experience social marginalisation that goes beyond age, gender and ethnicity.^15^

In this paper, we focus on social marginalisation resulting from homelessness, justice involvement, SU problems and pre-existing mental ill-health because these are core aspects of severe and multiple disadvantage and are often experienced together.^9 16^ The challenges faced by people experiencing these situations differ fundamentally to the general population and intersect with help-seeking in different ways. For instance, women experiencing homelessness face competing priorities (such as accommodation loss), may hide difficulties from authorities who have powers to intervene with their children, and experience exhaustion from navigating the system, leading them to cease seek help.^17^ People in custody can be deterred from help-seeking for MH or SU due to a perceived need to project strength, control and autonomy, in order to retain their status and related safety.^18 19^ Among those in prison, negative past experiences can lead to mistrust, a perception help is useless and fear of further negative experiences.^18 19^ Thus, this paper explores social marginalisation arising from experiences such as homelessness and justice involvement, rather than socio-demographic factors.

Applying social theory to attain a more nuanced understanding of the relational influences on help-seeking for MH and SU among people experiencing social marginalisation is essential given the disproportionate prevalence of MH and SU problems in these populations and the identified evidence gaps. Desire and ability to seek help involve personal and relational influences, including the attitudes, beliefs, knowledge, norms and resources in communities and peoples’ social networks. Existing theoretical models point to the relevance of these relational aspects when applied to help-seeking.^20 21^ However, these relational aspects remain under-explored compared with individual factors, particularly among people experiencing social marginalisation. This is important when considering the range of relationships people have and their embeddedness within communities with different cultures and social representations of help-seeking.^7^ Further, there has been limited application of social theory to explain the relational mechanisms behind help-seeking in populations experiencing social marginalisation,^19 22 23^ potentially impacting our ability to understand how and why help-seeking takes the forms it does among those most excluded from communities. Social capital provides an understanding of relational influences based on social connections,^21 22^ as opposed to approaches which focus more on individual or structural influences (eg, ^2124,26^).

Social capital can be considered the resources (eg, information, emotional and tangible support) available to an individual through their relationships. Social capital theory explains how the structure and nature of relationships, at micro (individual), meso (social network) and macro (community/societal) levels, enable or constrain behaviour through access to resources and information and the sharing of beliefs, attitudes and cultural norms.^27^,^29^ This enables potentially conflicting influences which might impact an individual to be disentangled. For example, SU might be actively encouraged among someone’s peers but stigmatised among their family and local community. Social capital has been further elaborated to consist of bonding, bridging and linking capital. Bonding capital refers to the social capital obtained through relationships within a group, whereas bridging refers to that obtained from relationships between groups.^29 30^ Linking capital is conceptualised as social capital obtained from relationships across different levels of a social hierarchy, such as between citizens and institutional providers of services.^31^ Social capital theory therefore provides a means to theorise the relational influences on help-seeking for MH and SU within social networks embedded in communities.

Evidence indicates a positive relationship between social capital and health, although different groups do not always benefit or benefit equally.^32^ Social capital can have positive or negative implications for a range of outcomes including health.^33 34^ There is a need for greater empirical examination of the mechanisms by which social capital has its effects and how this may differ between and within groups.^32^ To maintain good health, the influence of social capital on accessing help at a time of illness, through either facilitating or deterring help-seeking, is one potential mechanism that could contribute to better health outcomes for people experiencing social marginalisation.

If relational influences act through peoples’ social networks, empirical research is needed at this level. Social network analysis (SNA) takes an approach to research in which networks are a central concept of interest.^35^ SNA is well suited to examining social capital at the ‘meso’ level, that is, at the level of networks within communities (with micro representing study of the individual, such as their characteristics, and macro representing wider societal structures, such as law or policy). It presents a means to elucidate the mechanisms by which social capital may affect different outcomes, including help-seeking. SNA includes a range of methods to study structure, composition and dynamics of social networks in their situated context.^36^

There are two primary applications of SNA. The first involves analysing personal networks of individuals (eg, egocentric networks or ‘egonets’) by collecting quantitative and/or qualitative data about a person (ego), others to whom they relate (alters) and how alters relate to one another. The second involves analysing usually quantitative data on a whole network (sociocentric networks) of all people within a bounded community, such as a school, and identifying the connections between them. In both cases, ego and alter characteristics can be collected, and the nature of relationships ascribed different attributes (eg, supportiveness or conflict levels). Analyses can be descriptive, involve identifying patterns in behaviour or modelling to predict outcomes. SNA has utility for gathering and analysing data to empirically examine the relational influences on help-seeking for MH and SU and the mechanisms by which these operate.

There is limited literature that examines MH or SU help-seeking among people experiencing social marginalisation, especially that which considers relational over individual influences. Better understanding relational influences is essential to inform tailored approaches that enable those experiencing stark health inequities to seek, and ultimately access, help for highly stigmatised health needs. Social capital theorises how and why relationships in social context influence behaviour, indicating its potential utility for understanding influence on help-seeking that goes beyond individual or structural considerations. The growing application of SNA in health research, and its suitability for operationalising and empirically examining social capital, suggests SNA research may highlight key considerations for understanding help-seeking for MH and SU among people experiencing social marginalisation.

The broad academic and practice fields in which social capital and SNA are applied indicate a need for a multidisciplinary synthesis to minimise fragmentation in the evidence base. Using a scoping review, we aimed to establish current knowledge about: the utility of social capital theory to explain the relational influences on help-seeking for MH and SU in people experiencing social marginalisation and how SNA methods have been applied to study relational influences of help-seeking for MH and SU with people experiencing social marginalisation.

### Objective

To synthesise the literature applying social capital theory or SNA to understand relational influences on help-seeking for MH and SU among people experiencing social marginalisation.

### Review questions

1. How have theories of social capital been applied to understand the relational influences on MH and SU help-seeking among people experiencing social marginalisation?
2. How have SNA methods been applied to understand the relational influences on help-seeking for MH and SU among people experiencing social marginalisation and what methodological insights have been gained?

## Methods

We followed established scoping review methods^37 38^ and report this using the Preferred Reporting Items for Systematic Reviews and Meta-Analyses Scoping Review Extension (PRISMA-ScR).^39^

### Protocol and registration

We pre-published our protocol on OSF (www.osf.io/nfkmq).

### Patient and public involvement

This review is part of a larger study informed by a Patient and Public Involvement Group consisting of people with experience of justice-involvement. They have confirmed that understanding how to increase early help-seeking for MH and SU is important and will advise on the dissemination of review findings alongside wider study activities.

### Eligibility criteria

Eligible papers examined MH or SU help-seeking from any source among adults experiencing social marginalisation beyond sociodemographic characteristics, and applied either social capital theory or SNA. We did not limit context or date, but restricted language to English and our search to peer-reviewed sources.

We sought papers which focused on or discussed help-seeking for MH or SU among adults (18+) experiencing social marginalisation. We regarded help-seeking as an individual’s action(s) to try to elicit, or increase their levels of, help. Papers focused on people already receiving help without searching for additional help were excluded as these were not about seeking help.

We considered aspects of social marginalisation that went beyond sociodemographic factors. This included involvement in the criminal justice system (justice involvement), homelessness, pre-existing mental ill-health, problematic use of drugs or alcohol or gambling.

We included papers if they used social capital theory or applied SNA. While there are wider ways of thinking about relational influences on help-seeking, we focused on literature applying social capital theory or SNA approaches due to their growing application across fields of research, and to keep the review firmly based around relational influences.

Initial scoping searches identified a very limited literature applying social capital or SNA methods to MH or SU help-seeking when excluding papers where the marginalisation experience was solely related to MH or SU problems. Papers where marginalisation was solely related to MH or SU problems offered relevant findings and were included due to the prevalence of MH and SU problems among those experiencing marginalisation due to homelessness or justice involvement.^9 16^

### Information sources

We searched four databases to identify literature across health, social science, criminology and criminal justice: Criminal Justice Abstracts (CJA), EMBASE, SocINDEX and Web of Science (all databases).

### Search strategy

Initial searches for SNA or social capital theory *and* MH or SU in Web of Science produced a manageable number of publications for review. We thus proceeded in our full search without using search terms for help-seeking or social marginalisation, allowing us to take a nuanced approach to inclusion that accounted for variation in definitions and terminology. As a scoping review, we did not include an exhaustive range of types of social marginalisation, selecting problem SU (alcohol and drugs), problem gambling, mental ill health, homelessness and justice involvement due to their association with multiple disadvantage.^9 16^

The review team considered papers carefully for whether the phenomena studied could be interpreted as help-seeking for MH or SU. We determined this to be where a person approached another/others to discuss or disclose MH or SU concerns or where someone sought advice, support or help related to their MH or SU. We attempted to capture the inverse, where people were ‘not help-seeking’ by disengaging from or avoiding treatment and support. Consistent with scoping review methods, we did not use every possible term for MH and SU, but selected relevant terms in conversation with a subject librarian that would provide broad coverage.

In each database, we combined search terms for key concepts using Boolean operators. We searched title, abstract, keyword and, where available, subject headings/indexing. In Web of Science, we searched by ‘Topic’ (title, abstract and keywords). Table 1 shows the template search strategy, with the full search strategies included in the online supplemental file 1.

**Table 1:** Template search strategy

Questions	Key concepts	Key search terms
How have theories of social capital been applied to help-seeking for MH and SU among socially marginalised groups?	Social capital theorySocial network analysisMental healthSubstance useHelp-seekingSocially marginalised groups	“Social capital”OR“social network analysis”AND“mental health” or “mental illness” or “psychosis” or “schizophrenia” or “depression” or “anxiety” or “personality disorder” or “eating disorder” or “post traumatic stress disorder” or “suicide” or “suicidal”OR“Substance use” or “substance abuse” or “substance misuse” or “addiction” or “drug dependence” or “alcohol dependence”
What insights have been gained through applying SNA to understand help-seeking for MH and SU in socially marginalised groups?		

MH, mental health; SNA, social network analysis; SU, substance use.

We set up email alerts in Web of Science (which indexed all the included papers and had the largest number of results) to identify new publications as the review progressed until June 2024, reviewed the reference lists of included papers and met regularly to determine if additional searches were needed, although this was ultimately not required.

### Selection of sources of evidence

CC conducted the searches on 26–27th June 2023, imported results into reference management software^39^ to remove duplicates and imported de-duplicated results into Rayyan^40^ to facilitate team review. Email alerts for papers meeting our search criteria were checked and reviewed until June 2024 with the intention of adding further papers as they emerged.

In an initial calibration process, three reviewers (CC, DG, RK) independently screened the same 10% of citations at title and abstract level and discussed results to agree a consistent approach. Following this, the three reviewers screened all papers at title and abstract level with at least two reviewers screening each record. We met at the mid- and endpoint of title and abstract screening to discuss inclusions, exclusions and any areas for additional searches. Disagreements were resolved in a three-way discussion, producing a list of papers relevant for full text review.

The three reviewers independently read 10% of papers at full text to determine eligibility for inclusion as an initial calibration process. Following this, all papers were independently screened by at least two reviewers. We again met at the mid- and endpoint to discuss decisions and resolve any conflicts. We intended this stage to be iterative,^38^ but did not identify a need for further searches. We checked all references lists of included papers and relevant papers that were outside our scope.

### Data charting process and data items

Three reviewers (CC, DG, RK) piloted a data charting template before meeting to review the consistency in content and detail of the extracted data. Minor amendments were made to clarify whether a paper’s focus on help-seeking and social capital were explicit or incidental. We included papers that had incidental findings of interest but were not primarily focused on help-seeking/social capital/SNA due to the limited literature. We added extraction of sample size and study aim(s) and streamlined our recording of individual study results. Reviewers divided the remaining papers for data charting, with CC checking all extracts. It was not necessary to contact authors for further details. For each paper, we recorded the data detailed in table 2.

**Table 2:** Data items extracted

All papers	Year published Year data collected Location and country Population Sample size Sociodemographic characteristics (age, sex/gender, ethnicity, other) Aim and extent of help-seeking (implicit or explicit)
Social capital papers	Extent of focus on social capital (explicit or incidental) Level of social capital discussed (micro, meso, macro) Types of social capital explored (focusing on bonding, bridging and linking) Theoretical orientation of the paper Summary of any theoretical argument relating social capital to help-seeking Results relevant to help-seeking
SNA papers	Type of method (quantitative, qualitative, mixed methods) Social network type (eg, egocentric network/personal network, whole network) Network features studied (eg, centrality, homophily) Data collection and analysis methods Findings related to help-seeking or the utility of SNA

SNA, social network analysis.

### Critical appraisal

We anticipated we may find more theoretical papers discussing social capital, and because of our methodological focus on SNA, we elected to appraise the quality only of empirical SNA papers. As there are no validated critical appraisal tools for SNA, at least one reviewer appraised the quality of each paper using the Mixed Methods Appraisal Tool (MMAT), selected for its ability to support efficient appraisal of various study types.^41^ We were cognisant of potential limitations in elucidating methodological strengths and limitations of SNA designs and in this respect were testing the utility of MMAT for SNA.

### Synthesis of results

We synthesised the results using data summaries and visualisations, before developing a narrative synthesis addressing each question. For papers discussing social capital, we summarised, grouped and compared these according to the number and type of papers, publication dates and rate, and sample, sample demographics, country, focus on MH or SU (or both), theorists and types of social capital. For SNA papers, we summarised the number and type of study, sample, sample demographics, country, focus on MH or SU (or both), and methods applied. These were discussed as a wider team at the midpoint to capture impressions and inform the final synthesis and reporting.

## Results

### Selection of sources of evidence

**Figure 1:**
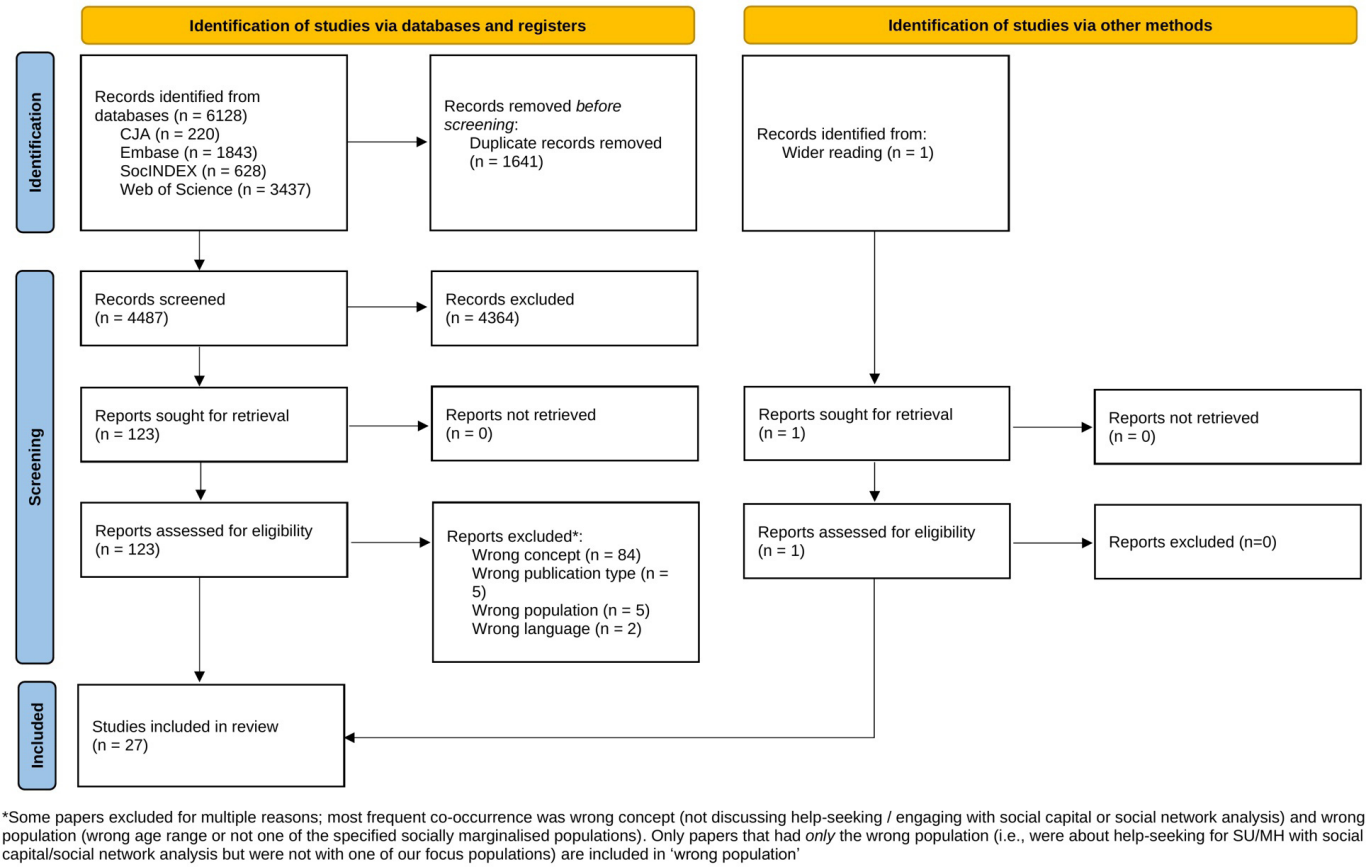
Preferred Reporting Items for Systematic Reviews and Meta-Analyses flow diagram. CJA, Criminal Justice Abstracts; MH, mental health; SU, substance use.

We identified and screened 4487 papers at title and abstract level and reviewed 122 at full text. We included 27 papers. Five were studies focused on help-seeking, of which four were identified in database searches and one from wider reading. The latter had not used ‘social network analysis’ as a term in full, thus not being identified in initial searches (see figure 1). An additional 22 papers were retained in the review due to incidental references to help-seeking. While help-seeking was not the focus, they provided useful synthesis material to better understand the phenomenon.

### Characteristics of sources of evidence

We summarise the characteristics of the included papers here and present our full data extraction in online supplemental table S1 (Social Capital Papers), and online supplemental table S2 (Social Network Analysis Papers).

#### Publication date

**Figure 2:**
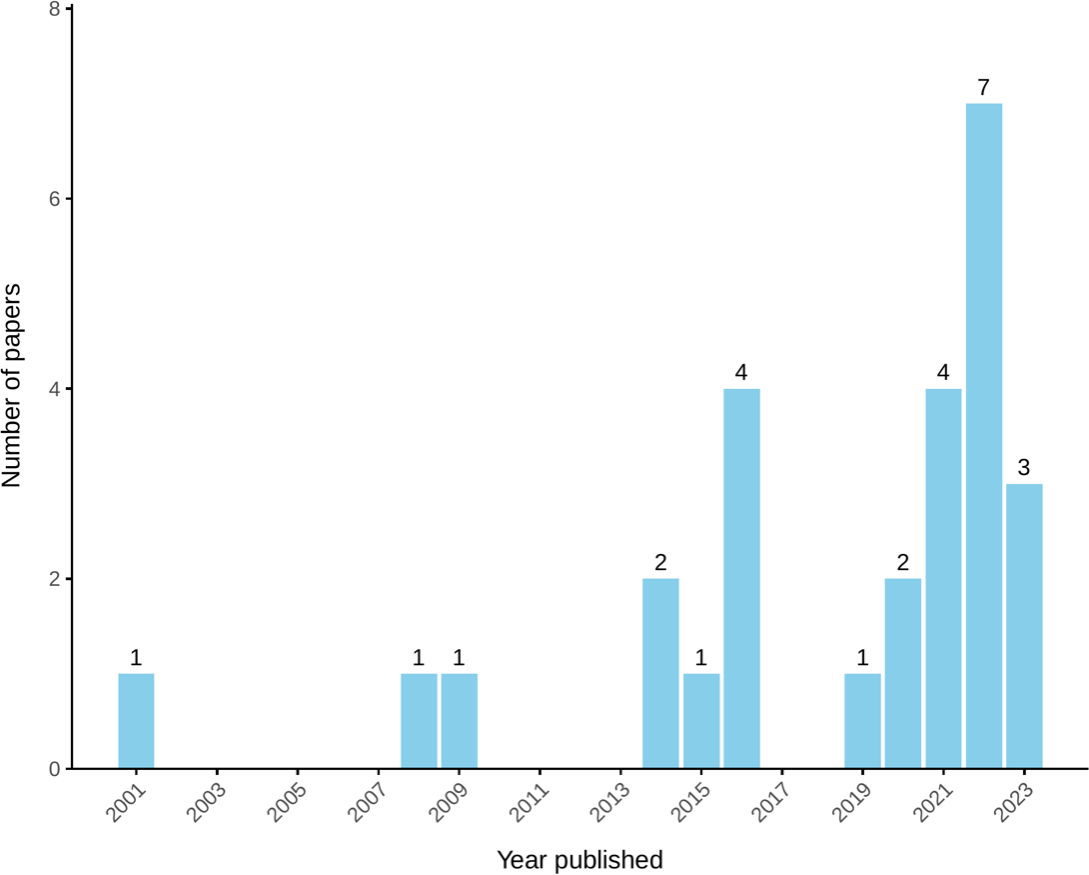
Numbers of papers published per year.

Papers were published between 2001^42^ and 2023.^43^,^45^ There has been a gradual increase in papers published that discuss relational elements of help-seeking for MH/SU among people experiencing social marginalisation (figure 2), although the vast majority only make incidental references to help-seeking.

#### Country

**Figure 3:**
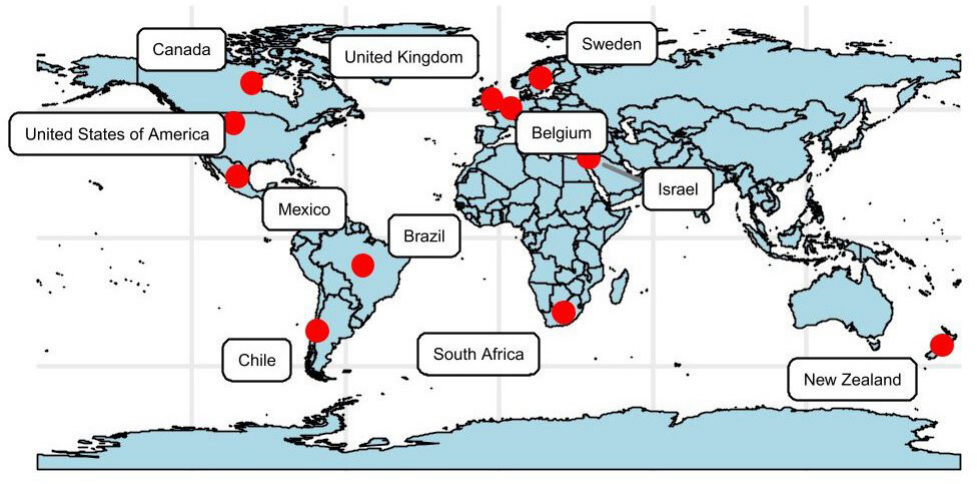
Countries represented in included studies.

High- and upper middle-income countries are represented (figure 3), with the largest number of studies from the USA (n=7). Two literature review papers potentially included participants from multiple countries,^46 47^one study was conducted online and therefore could include participants from multiple countries^48^ and one did not report the country where data were collected.^49^

#### Sample size

Sample size varied by study design from seven in the smallest qualitative study^45^ to 4211 in a whole network study that took data from internet posts.^48^

#### Demographics

With the exception of the literature reviews,^46 47^ most studies reported age, gender and ethnicity of their primary participants. Seven studies had a clear majority (over 60%) of men,^4243 45 50^,^53^ seven had a clear majority of women,^54^,^60^ seven were relatively equally split^4961^,^66^ and four did not report sex/gender of the sample.^44 48 67 68^

People aged 16 to 72 are represented. Five papers did not report the age of participants.^44 48 59 67 68^ Three papers examined the experiences of a specific ethnic group: African Americans in the USA,^63^ Orthodox Jews in Israel^51^ and Roma people in the Czech Republic.^44^ Ten papers did not report the proportions of people of different ethnicities, while the others included mixed ethnicity samples,^4450 54^,^57 62 65 68^ although for some this mix was minimal.^43 58 61^

SU descriptors were frequently provided.^4245 50 52^,^54 61 63 66^ Few papers provided MH descriptors.^56 58 64^

#### Type of marginalisation

Fulginiti *et al*^64^ and Oliver and Cheff^55^ sampled young adults experiencing homelessness. Timmer *et al*^47^ reviewed the literature on people with multiple disadvantage in contact with the criminal justice system. The remaining papers sampled people whose marginalisation experience was due to mental ill health (n=5) or drug use (n=19). However, most samples likely experienced multiple types of social marginalisation.

#### Issue help sought for

Two papers examined disclosing suicidality,^49 64^ one focused on approaching people to discuss health in the context of recent onset of mental ill-health,^56^ one studied advice-seeking about Buprenorphine for treating opioid dependence,^48^ and in line with our intention to capture ‘not help-seeking’, one paper discussed why people disengaged from SU treatment.^53^ The remaining papers incidentally reported on help-seeking within the wider findings. No studies used the term help-seeking.

### Critical appraisal within sources of evidence

Applying the MMAT^41^ to SNA papers provided an initial framework for assessing quality. While this allowed us to identify broad areas of strength and limitations, it provided little direction to the specific considerations for different types of social network studies. The main areas where we identified limitations were in clarity of reporting methods, particularly in how qualitative findings were synthesised and how multiple methods were integrated in a single study to reach conclusions. See online supplemental table S2.

## Results synthesis

The results of individual sources of evidence are presented in online supplemental tables S1 and S2.

### Application of social capital theory

Twenty-three papers referred to social capital. Only two studies were focused on help-seeking,^53 56^ of which only Perry and Pescosolido^56^ directly applied social capital in their work. The remaining 21 were focused on other phenomena but with incidentally relevant findings.

Most papers were qualitative, with two literature reviews, ^46 47^one mixed methods study^50^ and one quantitative study.^56^ Eighteen used or described social capital, while five only made a short reference to it.^52 54 58 59 66^

#### Theoretical orientations

Most papers cited the seminal works of Bourdieu,^27^ Coleman^28^ and Putnam.^29^ Perry and Pescosolido^56^ present the only detailed theoretical argument for the role of social capital in influencing help-seeking, in the context of mental illness, drawing from network theory of social capital^69^ and their own previous work.^22 23 70^

Six studies were framed using social capital theory, as elaborated by a particular theorist(s), but only incidentally referred to help-seeking.^50 55 57 63 65 68^ Five qualitative papers and one literature review provided a general description of social capital and key theorists, without any further framing of their study, and picked up relevant findings in the discussion.^4251 60^,^62^

Seven studies^44^,^4652 53 58 67^ referred to social capital within ‘recovery capital’, citing the work of Granfield and Cloud.^42 71 72^ Recovery capital draws on the work of social capital theorists but is a broader concept for explaining SU recovery that incorporates personal, physical and cultural capital. We only included papers that referred to the social capital component of recovery capital. Radcliffe and Stevens^53^ discuss reasons for not seeking help (treatment engagement/disengagement), but reference to theory was limited in the findings and discussion. Three papers did not provide a theoretical basis for their references to social capital.^54 59 66^

#### Forms and levels of social capital

No studies considered bonding, bridging, and linking specifically in relation to help-seeking. Six papers mentioned bonding, bridging and linking in relation to their data.^45 46 50 51 55 61 67^ Only Silva *et al*^57^ explicitly stated the levels of social capital of interest in their study. They considered the micro and macro levels, defining these as how individuals interact within groups and as how thoughts and behaviours influence network structures respectively. However, they do not apply this explicitly to understanding help-seeking.

#### Methodological insights gained through applying social network analysis (SNA)

Eight papers reported using SNA. Six used quantitative SNA: four focused on help-seeking,^48 49 56 64^ of which three adopted multilevel modelling.^49 56 64^ One SNA paper adopted mixed methods,^50^ and three used qualitative SNA.^43 57 65^ Two papers did not use SNA in full, instead using elements of it to guide data collection and coding of qualitative data respectively.^57 65^ We include them here as they engage at the relational level in their data collection and/or analysis.

One study used whole network SNA, taking data from online forums^48^ and analysing this statistically. Seven papers used personal network SNA.^43 49 50 56 57 64 65^ Three studies involved the completion of a network map during interview.^43 49 50^ Analytical approaches in the personal network studies varied, reflecting the different types of data and collection methods.

#### Conclusions about the utility of social network analysis (SNA) from paper authors

The authors considered qualitative SNA to be useful for understanding relationships and their functions,^43^ including relationships from which someone could seek help. Network maps were identified as a valued interview tool for achieving more depth in understanding relationships^43^ and aiding recall for achieving data completeness.^49^

Multilevel modelling was emphasised for its ability to generate novel findings precluded in traditional statistical analysis, by accounting for the unique contributions of individual characteristics, relationship factors and network features.^49 56 64^

The potential for ego perceptions of alters’ characteristics and relationship qualities to be subjective was reported as a limitation.^65^ However, it could be credibly argued that it is the ego’s perception that is of interest in understanding the relational influences on them.

Results from SNA studies could inform more nuanced network-based interventions that allow for relational level influences on individual behaviour to be considered within the wider social context.^50 65^

### Relational influences on help-seeking

#### Fear of losing social capital

Fear of losing social capital had an important influence on whether to initially seek help and continue to do so. People feared that being seen to be associated with a drug-using community, which would be highlighted by attending treatment, could result in a loss of conventional social capital given the associated stigma.^53 66^ In contrast, being known to have engaged with treatment or to have tried to move away from SU could risk the loss of alternative forms of social capital that marginalised communities depended heavily on. For example, losing the status that can be attained in the drug-using community^53^ or the protection and employment offered by drug gangs^54^ may disincentivise help-seeking.

#### Risks of high bonding capital

A consistent theme was the risk that high bonding capital (where people in a community are well connected to one another), in the absence of bridging and linking capital, could limit the ability to attain knowledge about resources and opportunities to seek help.^45^,^4750 61^ Bonding capital may be beneficial for help-seeking where relationships are with people perceived as likely to be helpful or who encourage help-seeking. However, high bonding capital could also deter help-seeking, for example, where there are strong norms around abstinence that a person may feel they cannot or do not want to attain, or where there is a risk of rejection if someone experiences a relapse.^45 50^ High bonding capital within tight-knit communities where there is ignorance, stigma and reluctance to acknowledge SU could be equally restrictive,^44 51^ as well as where there is strong bonding capital connected to SU.^53 54^ Most papers exploring this phenomenon were among people who use drugs. Among people with mental illness, a small number of people with relationships characterised by closeness, kinship or partner roles were most likely to be approached,^56^ indicating that bonding capital is still important.

#### Service providers as social capital sources

Service providers were seen as a source of bonding, bridging, and linking capital. Service providers were sometimes the only sources of bridging and linking capital in peoples’ lives and vital for accessing support and sustaining engagement with it.^45 52 55 61^ Service-facilitated opportunities to develop bonding capital with other people experiencing social marginalisation were sometimes valued.^61^ However, sometimes this was not achieved or was not desired by participants, for example, where they did not see value in building relationships with others struggling with their own difficulties.^52 53 62^

#### Selectivity/preferred sources of help

People selectively seek help from others when their relationships are characterised by care, helpfulness and support, availability, closeness, commitment, trust and companionship.^43 49 56 57 64 65^ This selectivity is evident in that people only approach a subset of alters for help.^49 56 64^ Among adults with mental ill health, disclosing suicidality or seeking health-related discussions with particular alters were associated with alter age, having previously disclosed to that alter, relationship type, closeness, social support provided in that relationship and shared experience of mental ill-health.^49 56^ Previous social support was associated with disclosure of suicidal thoughts to particular friends among young adults experiencing homelessness, both historically and at the time of experiencing suicidality.^64^ Among people accessing MH services, the people to whom someone had disclosed and intended to disclose to differed.^49^ This suggests that experiences of help-seeking from particular alters may inform selectivity around who is approached in the future.

There are contrasting findings across studies about the roles of alters (eg, family, friend, service provider) from whom people seek support. Among people with mental ill-health, Amadei *et al*^43^ found family were most commonly approached, with professional service relationships of secondary importance. Perry and Pescosolido^56^ refine family further, identifying that mother and partner were most likely to be approached, with professional services equally as likely to be approached as neighbours, siblings, friends and fathers. Young adults experiencing homelessness were more likely to approach friends,^55^ and homeless adults using crack cocaine preferred specialist services and used emergency care.^57^ Peer/mutual support was viewed positively for some people seeking MH or SU support, describing these as relationships and communities that embodied care and belonging.^43 50^ This was supported by findings that alters who shared an experience of mental illness had significantly increased odds of being approached for help.^56^ However, it is first essential to know who and where peers are and maintain good relationships with peer leaders to avoid ostracism,^66^ again highlighting a risk in strong bonding social capital. There was an intersection between friends and professional service providers, with friendship sometimes attributed to professionals, including religious leaders,^43^ raising the issue of defining roles clearly in network studies.

Irrespective of role, alters were only approached when they were anticipated to be understanding and honest.^42 52 62^ Further, some participants were concerned about burdening friends/family^42 62^ or not wanting to be seen to complain.^60^ Support can be vulnerable to change when the person/people providing support also experience social marginalisation and have limited resources of their own.^60^ Further, family and friends could be actively unhelpful, with professional services expertise being preferred.^55 57 58^

#### Trust

Only Perry and Pescosolido^56^ discussed trust held within the network, in line with considering this as a resource. They showed a greater likelihood of an alter being approached to discuss health matters where network level trust in physicians is higher. All other studies reflected an understanding of trust as either something held by the ego or in some cases as a property of an alter/relationship. Previous help-seeking experiences impacted on trust that other people were likely to be helpful, which influenced future help-seeking attempts.^50 51 55 57 62 66^ This may explain quantitative SNA results, which demonstrated selectivity and change in who was approached for help at different time points.^49 56^ Past negative experiences, harms or neglect by authority figures,^51 55 60^ and service providers specifically,^50 57 62 66^ could erode trust in relationships in general, as well as in specific relationships, and deter future help-seeking. For young people, this contributed to a desire to prove they did not need support or could live without it, presenting a psychological barrier to seeking help if and when needed.^55^ While the absence of trust could be a barrier, Kirst^65^ and Oliver and Cheff^55^ highlight the importance of trust and companionship in relationships for enabling someone to access resources mediated by relationships with peers and service providers. Palombi *et al*^67^ noted that hoping that help will be effective plays an important role, alongside fear of what a response may be, reflecting the concept of trust in relationships.

#### Network culture

The importance of network culture and its influence over time on forming norms, beliefs and attitudes towards help-seeking across a community has received limited specific empirical examination in marginalised groups. Over and above the characteristics of egos and alters, the average level of pro-health care attitudes across a social network, indicative of network culture, is associated with help-seeking in the context of mental illness.^56^ The impact of tensions between varying cultural norms within one person’s network is illustrated with an example from Chile, where Mapuche people could be encouraged or discouraged to go to psychiatric services or Machi healers by different members of their community.^68^

#### Societal level influences

Structural barriers to accruing social capital and influencing help-seeking were discussed to a very limited extent. Stigma, drug laws and policy that fail to recognise opportunities for harm reduction, and wider structures that embed social marginalisation on the basis of ethnicity, gender, sexual orientation and gender identity were all mentioned, but with limited elaboration of their relevance to help-seeking.^47 59 65^ African Americans may face additional barriers to help-seeking in the USA because their ethnicity places them in a position of lesser power,^47 63^ resulting in a lack of social capital that would enable access to resources and information to facilitate help-seeking.^47^

## Discussion

This review presents a summary and synthesis of current knowledge, across diverse disciplines, of the relational influences on help-seeking for mental health (MH) and substance use (SU) problems among people experiencing social marginalisation. Specifically, we examined applications of social capital theory and SNA.

### Summary of evidence

There is limited literature that uses social capital theory or SNA to examine relational influences on help-seeking for MH and SU among people experiencing social marginalisation. We identified few studies that sampled people experiencing homelessness,^55 57 64^ and the only paper discussing justice-involved people was a literature review.^47^ In the few studies explicitly focused on help-seeking, the term ‘help-seeking’ was not widely used. This may have implications for future reviews attempting to examine help-seeking for MH, SU or other needs, potentially resulting in few results. A priori theorisation and decisions about what the review team considers ‘help-seeking’ will be beneficial.

High bonding capital among people experiencing social marginalisation to others within minority communities, in the absence of bridging and linking capital, could deter help-seeking for MH and SU. This extends the evidence on the health risks of high bonding capital^32 34^ by highlighting that deterrence of help-seeking may be a mechanism by which social capital affects health. We noted two ways in which this mechanism may operate. First, help-seeking may present a risk of losing social capital in networks and communities where norms and culture do not support engaging with authoritative structures, or where there is network and community stigma towards people who have MH or SU problems, thus deterring help-seeking. Perceived stigma towards people in need of help, translating into internalised stigma, has been shown to negatively influence MH help-seeking.^73^ While the risk of losing social capital also applies to people who possess high levels of bridging and linking capital, arguably the risk is more pronounced for people who rely on being part of a close-knit community. Second, an absence of linking and bridging capital can reduce access to varied information^74^ about when help can and should be sought, what is available and how this might be experienced, leaving people without the information needed to decide to seek help.

Viewing social capital as the sum of resources within a network, having lower bridging and linking social capital could indicate a network holds fewer resources, and thus influences people to make deliberate decisions about from whom to seek help. There were references to both seeking out friends and family and avoidance of doing so, where people were conscious of not burdening others with limited resources. This is in contrast to evidence that people make greater use of weak ties and avoid stronger ties when disclosing sensitive personal circumstances.^75^ However, Small’s conclusions were developed from studies with people with a greater degree of social integration (university students), who may have greater access to weak ties through their social positionality, and did not examine highly stigmatised issues. Findings from our review indicate that relationship qualities, including closeness, may be particularly important for sensitive and highly stigmatised illnesses among people experiencing social marginalisation. The small volume of evidence identified in this review limits our ability to draw firm conclusions, and the importance of tie strength may warrant greater exploration in populations where there are low levels of bridging capital.

Selectivity about from whom help is sought was clear, with preferences about alters’ social roles varying between studies.^49 56 64^ However, relationship qualities were more important than alters’ respective roles. Specifically, those relationships characterised as caring and perceived as likely to be helpful were the ones that were approached. Further, the alters that participants did or would approach changed over time, reflecting the influence of past experience on where, when and from whom someone may seek help. This contrasts with less deliberative approaches to help-seeking for lower-level concerns among students^75 76^ and is more consistent with theories that help-seeking is a socially influenced, but still deliberative, action.^22 23^

Qualitative studies showed that at times, participants characterised professionals as friends, highlighting a need for a clear understanding of how people define the roles of alters in their networks, especially when measuring this in quantitative approaches. In the quantitative analyses, variables associated with help-seeking were identified, with some conflicting results. More studies that account for different experiences of marginalisation in different contexts but using the same variable operationalisation could progress understanding of the relational influences on help-seeking and the best ways to apply social capital and SNA to study these.

Trust is debated by social capital theorists regarding whether it is characteristic of relationships or a more generalised community-level asset and whether it contributes to, results from or is integral to social capital.^77^,^80^ Trust is an important relationship quality that enables access to social capital, and network levels of trust in practitioners were associated with help-seeking. Mistrust of authoritative services is a well-elucidated cultural norm within some communities that deters help-seeking, for example, among people in prison^18^ and women who use drugs.^81^ Trust in healthcare providers at an individual level varies across communities, partly explained by levels of community trust and community social capital.^82^ The importance of network (and community) culture and attitudes towards different sources of support has received little examination in groups who experience marginalisation by some communities/social groups, but who have strong bonding capital in communities sharing their marginalisation experiences.^44^

Structural factors were considered to a limited extent (eg, law and policy that fails to allow for harm reduction opportunities, sociodemographic characteristics). Future research that elaborates exactly how these macro factors influence help-seeking for MH and SU among people experiencing social marginalisation is required, particularly accounting for the intersecting experiences that further compound disadvantage.^83^ Better understanding structural influences can inform and enhance the impact of ‘Inclusion Health’ policies and practice^84^ to assist those in greatest need of MH and/or SU support.

SNA methods present a useful means of empirically examining help-seeking among people experiencing social marginalisation, through use of visual network mapping, the application of multilevel models and its potential for informing intervention development. Although SNA in health and social care research is increasing, the limitations in the wider evidence base are evident here, including the need to better understand relational influences on health-related behaviour, the mechanisms by which social networks have their effects and greater use of longitudinal data to understand change over time.^85^

### Limitations of the included studies

No studies used the term help-seeking specifically. For relevant papers to be identified for future reviews, authors of primary research may consider adopting consistent terminology or including help-seeking as a keyword.

We only quality appraised SNA studies and did not exclude papers on quality grounds. Our main observation was limitations in the clarity of reporting, particularly in how qualitative SNA findings were synthesised and where multiple methods were combined in a single paper. We included 27 papers and provide a broad overview of how social capital and SNA are applied; thus, our findings can be read with sufficient confidence for that purpose. Our synthesis of findings from the social capital studies should be interpreted with awareness that these papers were not quality appraised.

Most papers included participants who used drugs, and several included participants experiencing MH challenges. Although homelessness, sex work, justice-involvement and poverty were all represented, and there are significant overlaps between populations, there is clearly a need for research that examines the unique relational dynamics influencing help-seeking when people experience social marginalisation due to different and multiple experiences. Core demographics were not or could not be reported for some studies. Reporting precise sample demographics and characteristics is essential for future reviews that seek to take account of the intersecting experiences of social marginalisation including intersections between age, sex/gender, ethnicity or sexual orientation.

### Limitations of the review

We conducted the review using rigorous methods, pre-publishing our protocol and adopting PRISMA reporting standards. We searched in English and excluded papers where the full text was unavailable in English. This may have resulted in relevant literature in other languages being overlooked, particularly in non-Western contexts. We nonetheless identified studies from countries with different social and cultural dynamics across five continents.

While we focused on selected marginalisation experiences, there are likely to be implications for other populations, such as those marginalised by poverty or long-term unemployment, from minority ethnic groups, diverse sexual orientations or different gender identities. However, the distinct relational influences on MH and SU help-seeking in each of these groups and others should be examined, in addition to where people have intersecting experiences of marginalisation.

While scoping reviews often omit quality appraisal, we appraised SNA studies. It was possible to use MMAT, but it lacked detail for the interrogation of SNA elements. Development of a bespoke tool that can be used for studies adopting this method may be beneficial.

Not all full texts were screened by two independent reviewers as originally intended. At initial and interim team meetings, we determined that the level of consistency achieved between reviewers was sufficient to allow a single review of remaining papers. Additionally, extraction and synthesis were conducted by a single reviewer. Two reviewers at each stage may have added further rigour to the review.

Social capital is not the only social theory that may be relevant, and SNA is not the only way to study it. Future reviews could assess how other theoretical or methodological approaches have been applied to understand the relational influences on help-seeking for MH and SU among people experiencing social marginalisation.

## Conclusion

Social capital can be useful in understanding the relational influences on help-seeking for MH and SU problems among people experiencing social marginalisation. SNA presents a useful method for examining social capital across the networks of people experiencing social marginalisation. Further theoretical elaboration and empirical work are needed, particularly to test whether aspects of social capital influence help-seeking and whether this mediates or moderates health outcomes. Future work should take account of shared and distinct phenomena across different experiences of social marginalisation, to strengthen the conclusions that can be drawn and thus inform policy, practice and intervention development.

## Supplementary material

10.1136/bmjopen-2024-090349 online supplemental file 1

## Data Availability

Data are available upon reasonable request. All data relevant to the study are included in the article or uploaded as supplementary information.
