# Supplementary material for: Relational influences on help-seeking for mental health and substance use problems among people experiencing social marginalisation: a scoping review
Source: BMJ Open. 2025 Jun 4;15(6):e090349. doi: 10.1136/bmjopen-2024-090349 (PMC12142075; doi:10.1136/bmjopen-2024-090349)
Supplement: online supplemental file 1 [file bmjopen-15-6-s001.docx]

Supplementary file

Contents

[Search strategies 2](#_Toc190935774)

[Criminal Justice Abstracts via EBSCO Host 2](#_Toc190935775)

[EMBASE via OVID 3](#_Toc190935776)

[SocINDEX via EBSCO Host 4](#_Toc190935777)

[Web of Science 5](#_Toc190935778)

[Table S1: Social capital papers 6](#_Toc190935779)

[Table S2: Social network analysis papers 11](#_Toc190935780)

# Search strategies

## Criminal Justice Abstracts via EBSCO Host

| **#** | **Query** | **Limiters/Expanders** | **Results** |
| --- | --- | --- | --- |
| S3 | (S1 AND S2) | Expanders - Apply equivalent subjects Search modes - Boolean/Phrase | 220 |
| S2 | TI ( “Substance use” or “substance abuse” or “substance misuse” or “addiction” or “drug dependence” or “alcohol dependence” or “mental health” or “mental illness” or “psychosis” or “schizophrenia” or “depression” or “anxiety” or “personality disorder” or “eating disorder” or “post traumatic stress disorder” or “suicide” or “suicidal” ) OR SU ( “Substance use” or “substance abuse” or “substance misuse” or “addiction” or “drug dependence” or “alcohol dependence” or “mental health” or “mental illness” or “psychosis” or “schizophrenia” or “depression” or “anxiety” or “personality disorder” or “eating disorder” or “post traumatic stress disorder” or “suicide” or “suicidal” ) OR AB ( “Substance use” or “substance abuse” or “substance misuse” or “addiction” or “drug dependence” or “alcohol dependence” or “mental health” or “mental illness” or “psychosis” or “schizophrenia” or “depression” or “anxiety” or “personality disorder” or “eating disorder” or “post traumatic stress disorder” or “suicide” or “suicidal” ) OR KW ( “Substance use” or “substance abuse” or “substance misuse” or “addiction” or “drug dependence” or “alcohol dependence” or “mental health” or “mental illness” or “psychosis” or “schizophrenia” or “depression” or “anxiety” or “personality disorder” or “eating disorder” or “post traumatic stress disorder” or “suicide” or “suicidal” ) | Expanders - Apply equivalent subjects Search modes - Boolean/Phrase | 86,290 |
| S1 | TI ( "social network analysis" or "social capital" ) OR SU ( "social network analysis" or "social capital" ) OR AB ( "social network analysis" or "social capital" ) OR KW ( "social network analysis" or "social capital" ) | Expanders - Apply equivalent subjects Search modes - Boolean/Phrase | 2,138 |

## EMBASE via OVID

Embase <1974 to 2023 June 26>

1 social capital.mp. or social capital/ 6193

2 social network analysis.mp. or social network analysis/ 3001

3 "substance use".mp. or "substance use"/ 70073

4 "substance abuse".mp. or substance abuse/ 76105

5 drug misuse/ or substance misuse.mp. 13383

6 addiction/ or addiction.mp. 153427

7 drug dependence.mp. or drug dependence/ 78207

8 alcohol dependence.mp. or alcoholism/ 133979

9 mental health.mp. or mental health/ 401346

10 mental illness.mp. or mental disease/ 293832

11 psychosis/ or psychosis.mp. 149976

12 schizophrenia.mp. or schizophrenia/ 234078

13 depression/ or depression.mp. 852167

14 anxiety/ or anxiety.mp. 494771

15 "personality disorder".mp. or personality disorder/ 54470

16 "eating disorder".mp. or eating disorder/ 41607

17 post traumatic stress disorder.mp. or posttraumatic stress disorder/ 83078

18 suicidal ideation/ or suicide/ or suicidal behavior/ 102925

19 suicid*.mp. 161363

20 1 or 2 9129

21 3 or 4 or 5 or 6 or 7 or 8 or 9 or 10 or 11 or 12 or 13 or 14 or 15 or 16 or 17 or 18 or 19 2116872

22 20 and 21 1843

## SocINDEX via EBSCO Host

| **#** | **Query** | **Limiters/Expanders** | **Results** |
| --- | --- | --- | --- |
| S3 | (S1 AND S2) | Expanders - Apply equivalent subjects Search modes - Boolean/Phrase | 628 |
| S2 | TI ( “Substance use” or “substance abuse” or “substance misuse” or “addiction” or “drug dependence” or “alcohol dependence” or “mental health” or “mental illness” or “psychosis” or “schizophrenia” or “depression” or “anxiety” or “personality disorder” or “eating disorder” or “post traumatic stress disorder” or “suicide” or “suicidal” ) OR AB ( “Substance use” or “substance abuse” or “substance misuse” or “addiction” or “drug dependence” or “alcohol dependence” or “mental health” or “mental illness” or “psychosis” or “schizophrenia” or “depression” or “anxiety” or “personality disorder” or “eating disorder” or “post traumatic stress disorder” or “suicide” or “suicidal” ) OR KW ( “Substance use” or “substance abuse” or “substance misuse” or “addiction” or “drug dependence” or “alcohol dependence” or “mental health” or “mental illness” or “psychosis” or “schizophrenia” or “depression” or “anxiety” or “personality disorder” or “eating disorder” or “post traumatic stress disorder” or “suicide” or “suicidal” ) OR SU ( “Substance use” or “substance abuse” or “substance misuse” or “addiction” or “drug dependence” or “alcohol dependence” or “mental health” or “mental illness” or “psychosis” or “schizophrenia” or “depression” or “anxiety” or “personality disorder” or “eating disorder” or “post traumatic stress disorder” or “suicide” or “suicidal” ) | Expanders - Apply equivalent subjects Search modes - Boolean/Phrase | 181,460 |
| S1 | TI ( "social network analysis" or "social capital" ) OR AB ( "social network analysis" or "social capital" ) OR KW ( "social network analysis" or "social capital" ) OR SU ( "social network analysis" or "social capital" ) | Expanders - Apply equivalent subjects Search modes - Boolean/Phrase | 11,717 |

## Web of Science

TS=(“Social capital” OR “social network analysis”)

AND

TS=(“Substance use” or “substance abuse” or “substance misuse” or “addiction” or “drug dependence” or “alcohol dependence” or “mental health” or “mental illness” or “psychosis” or “schizophrenia” or “depression” or “anxiety” or “personality disorder” or “eating disorder” or “post traumatic stress disorder” or “suicide” or “suicidal”)

# Table S1: Social capital papers

| **STUDY INFO** | | | | | | | | **HELP SEEKING** | **HOW SOCIAL CAPITAL THEORY IS APPLIED** | | | | | |
| --- | --- | --- | --- | --- | --- | --- | --- | --- | --- | --- | --- | --- | --- | --- |
| **First Author** | **Country** | **Population** | **Sample size** | **Age range (mean)** | **Sex/ gender** | **Ethnicity** | **Other** | **Help-seeking focus** | **Aim** | **Focus on social capital** | **Level studied (micro/meso/macro)** | **Type studied** | **Theorists** | **Results relevant to help-seeking** |
| Anderson et al. (2021) | UK (Scotland) | SU: peer-workers | 10 | Range 31-53 (median 41) | 100% male | NR | Time in recovery, peer worker status | Incidental | Increase understanding of recovery capital and social identity using mixed methods social network analysis | Explicit | Terms not used | Explicit: Bonding and bridging | Framing: Putnam  Cite: Network scientists | High bonding social capital within both drug using and recovery networks. Many participants were previously unsuccessful in accessing SU support, at a time when their networks were more characterised by bonding capital with other people who used drugs/alcohol (link to help-seeking not explicit). Some sought the stability of addiction services whilst others viewed services as unhelpful (due to limited opening, a perception that they were ‘ticking boxes’, and offering unwanted solutions). |
| Boeri et al. (2016) | USA | SU: Active and former drug users | 29 | Reported as age bands: 18-29 and 30+ | 48% female, 52% male | 66% White, 31% African American, 3% Hispanic | Using 'hard drugs'. | Incidental | Understand how people with problematic drug use and low-socioeconomic status access social networks to gain positive social capital over the life course | Explicit | Terms not used | Explicit: all | Framing:  None  Cite: Bourdieu & others | Limitations in treatment programs in providing wider bridging and linking social capital. This may reduce their impact and attractiveness to people to sustain engagement. By encouraging the sharing of experiences, and facilitating bonding social capital, they could sustain engagement. Family and friends were important sources from which to seek informal help, but without providing bridging/linking social capital, the impact of treatment could be short-lived. |
| Brookfield et al. (2019) | Lit review | SU: Methamphetamine users | NA | NA | NA | NA | NA | Incidental | Understand how to tailor support for methamphetamine users through meta-ethnography of experiences of cessation, recovery, and relapse | Explicit | Terms not used | Explicit: Bonding and bridging | Framing: none.  Cite: Granfield & Cloud | Potential for strong bonding social capital acting as a constraint on actions for change and risking contagion of unhelpful behaviours. Social capital determines the options available to people using drugs, including access to support, information, guidance and resources. Social capital is not static. |
| Brown & Baker (2020) | UK (England) | MH: People who have received treatment | 32 | 22-67 (NR) | 47% female, 53 % male | NR | Occupational background, welfare benefit receipt, | Incidental | Explore recovery experiences beyond health and social care or focussed on symptom management | Explicit | Terms not used | Terms not used | Framing: none  Cite: Bourdieu & others | By engaging in activities that consolidated a more rounded identity, people accessed and provided support in natural contexts. This was more attractive than seeking professional help, which some reported finding paternalistic or unhelpful. Participants noted that available friends and family was a privilege not shared by all. They made strategic choices about from whom and when they sought help to avoid ‘bothering people too heavily’. |
| Cheney et al. (2016) | USA | SU: People using and not in treatment. | 51 | 18-61 (36.1) | 47% female, 53 % male | 100% African American | Previously received treatment | Incidental | Understand how lifestyle and social network changes facilitate access to social capital needed to change cocaine use patterns, by exploring factors underlying African American cocaine users’ decisions to reduce or quit cocaine use | Explicit | Terms not used | Terms not used | Framing: Bourdieu | African Americans, particularly outside urban conurbations, experience personal, cultural, and structural barriers to accessing help and may rely on existing personal networks for help to a larger extent. They relate this to Bourdieu’s ideas around the struggle for power, and their position in society that limits access to conventional social capital. |
| Granfield & Cloud (2001) | USA | SU: People dependent on drugs/alcohol | 46 | Range NR (38.4) | 40% female, 60% male | 96% White | Addiction length, time in recovery, class backgrounds | Incidental | Examination role of social capital in self-recovery from alcohol- and drug-use problems | Explicit | Terms not used | Terms not used | Framing: none  Cite: Bourdieu, Coleman, Putnam & others | Granfield focuses on recovery without formal treatment. Friends and family could be the only source of help to which participants felt they could turn, but the ‘sympathy credit’ was noted as something that could be exhausted when drawing on intimacy and the commitment towards them held by others. |
| Itzhaki-Braun & Gavriel-Fried (2022) | Israel | SU: People who formerly used drugs | 14 | 21-45 (26.9) | 100% male | 100% Ultraorthodox jews | Addiction length, time in remission | Incidental | Understanding pathways of ultraorthodox Jews into and out of SU | Explicit | Terms not used | Explicit: bonding (minor reference) | Framing: none  Cite: Coleman, Putnam & others | Participants had felt unseen and unheard (rejected and ostracised) which reduced trust in others to offer support if asked, and prevented them asking for help. This was in the context of communities with strong bonding capital and limited knowledge and acceptance of SU that influenced the capacity and will of alters to know what to do and provide that. |
| Kirst (2009) | Canada | SU: people who use drugs | 80 | Range NR (42) | 48% female, 52% male | NR | Income, education level | Incidental | Understand how social capital can influence risk and health behaviours of people who inject drugs and smoke crack | Explicit | Terms not used | Terms not used | Framing: Coleman  Cite: Bourdieu, Putnam & others | Need for trust and companionship in relationships to facilitate access to/seeking of drug related advice. Services could erect barriers that deterred help-seeking, such as restricted hours. Harm reduction services were limited in what they could offer when in the context of restrictive public health and drug policies. These barriers to access may render the act of help-seeking too burdensome for people (not explicitly linked to social capital). |
| Myers et al (2016) | South Africa | SU: alcohol and drug users | 23 AOD users, 14 SPs | 16-21 (18.7) | AOD users: 100% women. SPs: 64% female, 36% male | AOD users: 52% Black African, 48% ‘coloured’ | Marital status, employment status education level, weekly polysubstance use   SPs: 5 health, 4 social welfare, 4 AOD treatment, 1 law enforcement | Incidental | Understand how the environment of poor young women who use alcohol or drugs informs access to and use of health services | Incidental | Terms not used | Terms not used | Framing: none  Cite: none | In the context of poverty and gang-embeddedness, there was a reliance on drug use, gang involvement and related activity for social capital and ultimately survival. This led to a reluctance to seek help for SU, as recovery may disrupt the income sources and status. There was a sense of hopelessness about seeking help, as participants were sceptical that anything could be achieved when returning to their context. |
| Oliver & Cheff (2014) | Canada | SU: homeless young women | 8 | 15-21 (18.4) | 100% women | 25% Caucasian, 13% West Indian, 13% South Asian, 13% Ojibwe, 13% Native Canadian, 13% Congolese, 13% Metis, | Sexual orientation, education level | Incidental | Explore how young women experiencing homelessness form attachments and accrue social capital outside of the nuclear family unit | Explicit | Terms not used | Explicit: all | Framing: Putnam  Cite: youth theorists | Participants felt alienated from support due to a distrust of providers, often based on past experiences. A trusted professional contact could act as a source of bridging social capital, by encouraging and facilitating help-seeking from other sources. In considering the distinct experiences of young people, an individualist narrative emerged as a barrier to help-seeking. The participants felt a need to ‘make it on their own’ and devalued social connections, potentially due to their age and need to assert their autonomy, but also due to repeat experiences of being hurt by others. |
| Ondrasek et al. (2023) | Czech Republic | SU: Roma people | 8 | NR | NR | 100% Roma | NR | Incidental | Explain high rates of self-recovery among Roma people who use drugs, within the socio-cultural context | Explicit | Terms not used | Terms not used | Framing: none  Cite: Granfield & Cloud | Cultural norms in the minority Roma community in the Czech Republic can prevent help-seeking. Norms include more acceptance of SU, little interest in treatment services, less knowledge about safe and unsafe SU, mistrust of majority institutions and concerns about risk of losing contact with family. Often SU, when it reaches a level perceived as problematic by the community, collective decisions to intervene are made. Intervention is imposed, using the strong bonding capital within the community to coerce and monitor behavioural change. |
| Palombi et al. (2022) | USA | SU: people in recovery | 64 | NR | NR | Over 85% white (only focus group level data given) | Drug court participation, city/township population, household income | Incidental | Examining the construct of social capital for people in short- and long-term recovery from substance use problems living in rural communities | Explicit | Terms not used | Explicit: all | Framing: None.  Cite: Granfield & Cloud | Participants held hope for help working, but fears about what a response may be that could prevent them seeking it. Some people initially mandated to attend could come to an appreciation of being held to account. Others were encouraged by a partner to seek help. |
| Perry & Pescosolido (2015) | USA | MH: People with a recent onset of mental illness | 171 | 16-72 (30.6) | 64% women | 73% White, 27% Black | Diagnosis, psychiatric symptoms, education, living situation, employment status | Explicit | Examining factors associated with activating social toes for health-related discussion | Implicit | Terms not used | Terms not used | Framing: Lin (not explicit).  Cite: others | Regular interaction partners = mean 15.5. Network partners with whom health issues are discussed = mean 3. Sample mostly had close ties (mean 2.64/3) with moderately high trust in physicians (mean 2.34/3). 20% of all alters are selected as health discussants suggesting selectivity.   Ego age and higher levels of psychiatric symptoms associated with increased odds of health discussion. Among alters, age, having a mental illness and having first suggested seeking help were associated with increased odds of health discussion. Relationships characterised by closeness and frequent contact more likely to be activated, those with conflict/hassles/problems less likely. Mother and health professional more likely to be approached than friend or partner. A child or co-worker less likely than a friend. Greatest likelihood of approach is mother or partner. Health professional no more likely that father, sibling, friend or neighbour. Alters in larger networks less likely to be approached.  Networks with higher levels of physician trust had greater odds of seeking help. |
| Pouille et al. (2021) | Belgium | SU: People in recovery - migrants and ethnic minorities | 34 | 18-60 (38) | 12% women, 78% male | Ethnicity NR (country of origin) | Range of substances, migrant generation | Incidental | Explore experiences of migrant sand ethnic minorities in recovery from problem substance use; personal, social, and community recovery resources that facilitate recovery; and elucidate barriers to recovery | Incidental | Terms not used | Terms not used | Framing: Granfield & Cloud | Participants described the people they could count on for help as understanding and honest, and it was these family members, partners, friends and peers they turned to. Some participants sought help from a religious deity. Participants described avoiding services when they thought there would be easily available substances, and some only made contact through an existing service. |
| Radcliffe & Stevens (2008) | UK (England) | SU: people who dropped out of treatment and service providers | 53 drug users, 14 treatment staff | 19-50 (Mean NR) | 26% women, 74% men | 75% White British, 8% Black British, 9% Mixed Heritage, 2% Asian British, 2% Traveller | Receipt of welfare benefits, employment status, time in prison for drug-related offences, drug treatment services mandated by court order. | Explicit | Exploring the relationship between shame and stigma, and engaging in treatment services | Explicit | Terms not used | Terms not used | Framing: none  Cite: Granfield & Cloud | Treatment regimes risked exposing drug user status to others, thus threatening loss of social capital. Fear of losing social capital contributed to disengagement from treatment as heroin use allowed greater concealment and flexibility. Engaging with treatment tarnished the participants’ sense of identity, associating them with ‘other’ drug users. This also risked their social capital, regardless of how much conventional social capital they had. Social capital exists among the drug using community. Participants expressed ambivalence towards this, but sustaining this social capital could conflict with treatment engagement. Service providers identified that status and identity associated with drug use was a draw to people with few other avenues to accrue social capital, and which could prompt disengagement. |
| Rijnink et al. (2022) | Aotearoa New Zealand | SU: people who inject drugs | 13 PWID   1 staff member | 30-51 (mean NR) | 43% female, 57% male | NR | Opioid substitution therapy status | Incidental | Explore experiences of people who inject drugs who rely on a needle exchange mobile outreach service | Incidental | Terms not used | NA (incidental social capital only) | Framing: none  Cite: none | In disaster and emergency situations there may be a perception, or reality, that help for SU is not there to ask for. Participants described needing to know who key peer contacts were and that they could help, and the importance of keeping good relations so that they did not lose out when others were favoured. Barriers to seeking help were the judgement experienced and fear of reporting by pharmacy staff, and the need to protect one’s identity and social status by not being seen accessing support. |
| Silva et al. (2021) | Brazil | SU: Homeless people who use crack cocaine | 17 SUs, 4 staff (+ethnography) | 22-53 (mean NR) | 65% female, 35% male | 94% Black or Brown | Monthly income | Incidental | Analyse characteristics of social support networks of individuals who used crack cocaine and supported by a Brazilian health program for people living on the street | Explicit | Explicit: Micro/macro | Terms not used | Framing: Lin  Cite: Lin | Participants looked for help from specialist providers over psychiatric or family support. The important characteristics were friendliness. They also relied on emergency services. |
| Skogens & von grief (2020) | Sweden | SU: young adults post treatment | 21 | 25-33 (29) | 76% female, 24% male | NR | MH problems, relationship status | Incidental | Examine how recovery over five years was related to former alcohol and drug problems, other problems and processes of change | Incidental | Terms not used | NA (incidental social capital only) | Framing: Granfield & Cloud.  Cite: Bourdieu & others. | Through engaging in treatment to resolve SU, some participants identified unmet MH needs. Recognition of previously obscured or un-acknowledge mental ill-health led to seeking MH support. Participants wanted to be taken seriously by people with MH expertise, seeking support outside the SU recovery community. The support most often desired was more ‘ordinary’ life support, that could be filled through social capital. |
| Smith et al. (2023) | UK (England) | SU: patients in harm reduction service | 7 | Early 20s to late 40s | 29% female, 71% male | NR | Peer mentor status, time in recovery services | Incidental | Explore experience of recovery from problem substance using captured images and descriptions | Explicit | Terms not used | Explicit: Bonding and bridging | Framing: none  Cite: Granfield & Cloud | Participants described isolation from potential sources of support, feeling alienated from any source of community or trusted confidents they could ask for help. Some experienced relief from 12-step-commuities where they could identify with others. Fear of relapse motivated continued engagement. Others expressed reluctance to reach out to people experiencing SU, and could experience alienation and exclusivity in 12-step communities if they could not relate to the group. As they were already using services, workers encouraged help-seeking for other issues. |
| Timmer et al. (2022) | Literature review | People experiencing multiple disadvantage | NA | NA | NA | NA | NA | Incidental | Develop an integrated theory of health care utilisation for justice involved people experiencing multiple disadvantage | Explicit | Terms not used | Terms not used | Framing: none  Cite: Lin, Bourdieu, Putnam | Argues that people released from custody (and experiencing multiple disadvantage) possess limited social capital and thus may lack knowledge about, and ability to navigate, the healthcare landscape to access care. This is exacerbated for racialised groups and potentially other minoritised identities, because of the intersectional disadvantages that impact accrual of social capital. They draw on a range of literature to propose this hypothesis and argue for the need for further testing of this model. They highlight the potentially reduced network size and thus fewer people to ask for support, return to areas where resources are limited, and stigma and racial discrimination as barriers. |
| Urada et al (2021) | Mexico | SU: female sex workers | 195 for quantitative part. Interviews with 16 professionals. Focus groups with 45 women | NR | 100% Female | NR | NR | Incidental | Understand if assets-based community developments make a difference to the lives of sex workers. | Incidental | Terms not used | NA (incidental social capital only) | Framing: none  Cite: none | Government providers present a negative view of the potential for help-seeking among women who use drugs, suggesting that people don’t ask for help with SU, that they don’t want help, and that they know and are fearful of societal rejection that can lead them to avoid going out. Potential of community empowerment initiatives to facilitate building of social capital in under-served areas or for people experiencing marginalisation. |
| Wiencke (2022) | Chile | MH: patients, and their relatives, neighbours, Mapuche healers, and psychiatric staff | 27 | NR | NR | NR | NR | Incidental | Learn about how social capital impacts people in mental health treatment | Explicit | Terms not used | Terms not used | Framing: Putnam  Cite: Putnam | There are conflicts between the psychiatric and traditional approaches with churches having a strong influence and some churches actively discouraging consultation of traditional healers (machis). Although staff thought confusion may be a barrier, this was not seen patient interviews. In one example case, the person seeks help first from the machis in line with his beliefs, before family members enforced a psychiatric consultation. Other patients experienced pressure to stop accessing machi. |
| Woodall & Boeri (2014) | USA | SU: women who use/formerly used methamphetamine | 30 | 19-51 (mean NR) | 100% Female | 87% White, 7% Latino, 3% African American, 3% American Indian | Income, employment status | Incidental | Examine how low-income women using methamphetamine the suburbs access resources, and how resources and networks impacts choices and outcomes | Explicit | Terms not used | Cited but not used | Framing: none.  Cite: Bourdieu, Coleman, Putnam & others | For some women, seeking help relied on the material and practical support of others. This could be from people with their own difficulties, or a single person, that left a woman vulnerable should the relationship change. Women stopped looking for help after attempts had been unsuccessful. They grew used to having needs met in other places, which may or may not be healthy options. There was reluctance to talk about failed access attempts or lack of resources for fear of being seen to complain. |

Abbreviations: AOD = alcohol or drugs, MH = mental ill-health, NR = not reported, SPs = service providers, SU = substance use

# Table S2: Social network analysis papers

| **STUDY INFORMATION** | | | | | | | | **HELP-SEEKING** | **INSIGHTS GAINED THROUGH SNA** | | | | | | | **QUALITY** |
| --- | --- | --- | --- | --- | --- | --- | --- | --- | --- | --- | --- | --- | --- | --- | --- | --- |
| **Reference** | **Country** | **Population** | **Sample size** | **Age range (mean)** | **Sex/gender** | **Ethnicity** | **Other** | **Help-seeking focus** | **Aim** | **SNA type** | **Network features** | **Data collection** | **Data analysis** | **Results relevant to methods** | **Result relevant to help-seeking** | **MMAT** |
| Amadei et al. (2023) | Brazil | MH: Patients | 16 | 22-59 (44) | 38% female, 62% male | 81% white, 13% brown, 6% black | Marital status, living arrangements, religion | Incidental | Analyse the structural characteristics, functions and attributes of the bonds in the users of the psychosocial care centre | Personal network  Qualitative | Size  Density Homophily Alters role Tie function | In person: Interview and network maps. File reviews. Institutional record review | Merging of network maps | SNA useful for understanding relationships and their functions (inc. help-seeking). Co-producing network maps a useful strategy. | People sought out relationships characterised by care, help, support, availability and commitment. Family were most commonly approached with professional service relationships second. Community mutual support was viewed positively, though not explicitly for MH support. They were experienced as places of care and belonging. There was an intersection between friends and service providers, with friendship sometimes attributed to professionals/religious leaders. | Lacks clarity in reporting analysis and how the qualitative data were integrated with the maps at individual and group level. |
| Anderson et al. (2021) | UK (Scotland) | SU: peer-workers | 10 | Range 31-53 (median 41) | 100% male | NR | Time in recovery, peer worker status | Incidental | To expand the conception of recovery capital and social identity | Personal networks  Mixed methods | Size. Density. Closeness. Homophily. Constraint. Transitivity. Betweenness centrality. Alters influence (negative/ positive). Network size. Network density.  Closeness (and mean closeness for network-level).  Homophily (E-I Index measured on network-level). Constraint. Transitivity. Betweenness centralisation. | In person: Interview and network maps. | Descriptive statistics. Variables converted to individual-level attributes for T-test and Z-test. Thematic analysis. | Demonstrated change in networks over time of transition (recovery), which could inform optimal intervention planning. The relational focus alone may omit insights into structural factors | People attempting to maintain abstinence could experience guilt in relation to their social networks where others continued to use substances, implying seeking support could be more difficult in this context, A participant highlighted how prison had been an opportunity due to forced abstinence and access peer support indicating that help-seeking may be more difficult when services or peer support are not visible. The dense networks of people in recovery mirrored those of people still using substances. In the former the structure could be supportive in maintaining abstinence (and potentially accessing help, whilst the latter could both support and restrict recovery efforts. | Small sample and use of retrospective accounts of previous networks may result in recall bias. Limited details on the alter characteristic.  Qualitative components are more robust than the statistical comparison. |
| Fulginiti et al. (2016) | NR | MH:  Outpatients with SMI | 30 egos,  438 alters | Range NR (47.7) | 57% female, 43% male | 36.7% Non-Hispanic White, 46.7% Latino, 10% Multiracial, 6.7% African American | Marital status | Explicit | Describe patterns of suicidal disclosure and "examine associations between individual-level (e.g., social support, stigma) and suicidal disclosure and relational level factors (e.g., availability, closeness, homophily) and suicidal disclosure." | Personal  Quantitative | Size  Alters roles | In person: Interview and network maps | Bivariate analysis. Multi-level models (alters level 1, egos level 2) | Multi-level modelling permitted novel understanding of disclosure practice taking account of individual and relational level factors  Network maps assisted recall for completeness. Method could strengthen development of accurate (suicidal disclosure) profiles to inform treatment planning | 77% had disclosed and 100% intended to disclose.  People disclosed to only a proportion of their social networks (14%) and intended to disclose to 23%. Roughly 1/3 alters previously disclosed to would not be approached again, whilst 15% of network members were identified as new potential alters for disclosure (selectivity and change over time).  Of individual factors, ethnicity remained a significant predictor of disclosure in multi-level models.  Of relational factors prior disclosure, relationship type, relationship closeness, and all types of social support remained associated with disclosure in the multi-level models. | Methods detail is thin: unclear how the sample were recruited. Modelling description is unclear. |
| Fulginiti et al. (2022) | USA | MH: homeless young people | 527 egos, 1318 alters | Range NR (21.0) | 33.1% female, 55.3% male, 11.7% gender minority | 22.9% Non-Hispanic White, 34.4% Black, 18.2% Latino, 24.5% Mixed/Other | Sexual orientation, education, homelessness duration, Traveller status, psychosocial variables, help-seeking variables | Explicit | Understand the prevalence and correlates of suicide-related disclosure in YAEH friendship networks at individual and relational level, considering concurrent and retrospective disclosure | Personal  Quantitative | Size. Friend types. Friend genders. Homophily (gender identity and sexual orientation). Contact frequency. Social support (tangible and informational/emotional). | In person: survey via laptop (self-administered or assisted) | Bivariate multilevel analysis. Multivariate multi-level models (alters level 1, egos level 2) | Finding relational level variables that can be modified to improve access to support overcomes the inconsistent individual level findings in other research | Average friend network size was 2.5.  30% had disclosed to a friend in their lifetime. Of these, 45% made this during crisis, and 55% in retrospect. 21% of friends were disclosed to, and of these 45% were approached during crisis and 55% retrospectively (selectivity).  Lifetime and concurrent disclosure were associated with lifetime history of unmet mental health needs and having friends who reliably offered social support (tangible and emotional/informational).  Young people who identified as transgender or gender non-conforming were more likely to disclosure current suicidal thoughts.  Post hoc analysis to compare friends with other relationships (family, provider, other) found no significant differences in disclosure patterns. | Well presented |
| Garcia et al. (2022) | Online | SU: People who use/formerly used opioids | 4211 users  (202 posts (44,103 comments) | NR | NR | NR | NR | Explicit | 1) identify and describe advice-seekers on Reddit for buprenorphine-naloxone use using text annotation, social network analysis, and statistical modelling techniques. 2) predict advice-seeking | Whole  Quantitative | Total degree. Eigencentrality. Closeness. Authority. Hub | Online: Webscraping tools used to extract relevant posts | Descriptive statistics. Mann Whitney-U and ChiSq tests with post-hoc comparisons (Benjamin-Hochberg). Generalised Linear Models | Combining individual and network factors produced the best fitting model for predicting advice-seeking in forum users | Advice-seeking posts were associated with people using (rather than formerly using) Buprenorphine.  Advice seeking was associated with having fewer and less close social connections.  Combining network and individual characteristics were better indicators of advice-seeking than either alone. | Well presented in terms of approach, although sample representativeness is unclear. Analysis is logical. Supplementary data presented reflecting good open science. |
| Kirst (2009) | Canada | SU: people who use drugs | 80 | Range NR (42) | 48% female, 52% male | NR | Income, education level | Incidental | Understand how social capital can influence risk and health behaviours of injecting drug users and crack smokers - comparative examination | Personal  Qualitative | Trust. Reciprocity. Companionship. Norms. Exchange of health information. | In person: Interview | Coding sensitised by the literature followed by development of themes. | Can demonstrate the complexities of social capital and the structural factors that influence decision making | See social capital section. | Well justified questions and data collection justified. Analysis lacks clarity about its deductive/inductive nature and the way the networks were analysed is not elaborated – but presents a coherent account of the issues identified. |
| Perry & Pescosolido (2015) | USA | MH: People with a recent onset of illness | 171 | 16-72 (30.6) | 64% women | 73% white, 27% black | Diagnosis, psychiatric symptoms, education, living situation, employment status | Explicit | 1) Identify how ego, alter, relationship and network characteristics impact tie activation for health matters during early stages of mental illness.  (also to assess impact of this on recovery - not relevant to this review) | Personal  Quantitative | Size Proportion of women Proportion of kin  Closeness  Trust in physicians | In person: interview | Identifying characteristics associated with activation health discussion ties using multilevel model (random intercepts) with alters at level 1 and egos at level 2. | Allows analysis at multiple levels (ego, alter, and network) | See social capital section | Well reported with clarity on limitations |
| Silva et al. (2021 | Brazil | SU: Homeless people who use crack cocaine | 17 SUs, 4 staff (+ethnography) | 22-53 (mean NR) | 65% female, 35% male | 94% black or brown | Monthly income | Incidental | Explore social support networks | Personal  Qualitative | Number of people/institutions participants’ network. Components linked to. Interaction type. Relationship strength/extent. Characteristics related to reciprocities. Boundaries from a group | In person: Interview. Ethnographic observation. Focus group. | Deductive coding considering micro and macro factors Graphic representation of network maps  Content analysis | NR | Participants looked for help from specialist providers over psychiatric or family support. The important characteristics were friendliness. They also relied on emergency services. | Overall the paper is difficult to follow in terms of what was collected and how it was analysed in relation to the SNA aspect of the study |

Abbreviations: ChiSq = chi-squared, MH = mental ill-health, NR = not reported, SU = substance use
