# Supplementary material for: Relational influences on help-seeking for mental health and substance use problems among people experiencing social marginalisation: a scoping review
Source: BMJ Open. 2025 Jun 4;15(6):e090349. doi: 10.1136/bmjopen-2024-090349 (PMC12142075; doi:10.1136/bmjopen-2024-090349)
Supplement: Supplementary file 2 [file bmjopen-2024-090349.reviewer_comments.pdf]

PEER REVIEW HISTORY

BMJ Open publishes all reviews undertaken for accepted manuscripts. Reviewers are asked to complete a checklist review form and are provided with free text boxes to elaborate on their assessment. These free text comments are reproduced below.

ARTICLE DETAILS

Title (Provisional)

Relational influences on help-seeking for mental health and substance use problems among people experiencing social marginalisation: a scoping review

Authors

Connell, C; Griffiths, Dave; Kjellgren, Richard; Greenhalgh, Jess

---

VERSION 1 - REVIEW

**Reviewer**	**1**
**Name**	**Lorant, Vincent**
**Affiliation**	**Université catholique de Louvain, Institute of Health and Society**
**Date**	**28-Aug-2024**
**COI**	**No competing interest.**

This study performs a scoping review about helpseeking behaviour of marginalised people with MI or with substance use disorder. In particular the authors investigate the way social capital is addressed and conceptualized in the published studies. The authors objective is to review the literature on social capital theory and SNA as applied to helpseeking behaviour for people with MI/SU.

I have some comments for this paper.

First, I am unsure about the overall objective "to synthetize the literature on social capital or SNA as applied to HSK for MH/SU among people experiencing social marginalisation". In the introduction, top of page 7, the authors emphasize the need for "better understanding of the relational influences of HPSK(...)". Then, on the same page, the authors shift to social capital and later on page 8 to SNA. If the authors are interested into the relational influences of HPSK then why not word the objective with such a wording "relational". Social capital is not the only way to conceptualize and address relationships. Similarly, SNA is more of a perspective then a concept so using SNA in the RQuestion makes the question more fuzzy

conceptually. There is a gap between the motivation of the review and the way the RQ is framed.

Second, I missed several key papers that are quite well known in this domain, having used SNA for people with MI. Notice that by definition people with severe mental illness are quite marginalized and I thus wonder whether adding the criteria of marginalization made sense at all. Here are a few studies I am missing and which match the selection criterial but I am sure that I could identify others. So my impression is that this scoping review has a rather restricted scope. It could be that scoping review is too specific. The criteria are a bit ambiguous: for example, it combines two criteria for the outcomes (MI/SU) and social marginalisation. Anyway, I think the papers mentioned below should have been included as they match the eligibility criteria mentioned on page 10.

-Sweet, D., R. Byng, M. Webber, D. G. Enki, I. Porter, J. Larsen, P. Huxley and V. Pinfold (2017). "Personal well-being networks, social capital and severe mental illness: exploratory study." British Journal of Psychiatry.

-Wyngaerden, F., P. Nicaise, V. Dubois and V. Lorant (2019). "Social support network and continuity of care: an ego-network study of psychiatric service users." Soc Psychiatry Psychiatr Epidemiol.

-Degnan, A., K. Berry, N. Crossley and D. Edge (2023). "Social network characteristics of Black African and Caribbean people with psychosis in the UK." Journal of Psychiatric Research 161: 62-70. She has another paper in SPPE as well.

Third, social network analysis and social capital are eligibility criteria but these terms are not on the same level: social capital is a concept where SNA is a perspective or a group of methods. So they don't align.

Results

From the middle of page 15 to page 21 we are given a long description of the study features, but this is a long and somewhat boring section that do not provide much information regarding the RQ. For example almost one page is devoted to describe the timing and location (including a map!!) of the studies and the information given in the figures duplicate somewhat the text. Is all this much necessary ? In a way, the interesting stuff begins on page 21. So I would strongly suggest to downsize this first descriptive section.

Theoretical orientations- on page 26, "in addition to Perry and Pescosolido 2015", this § seems to including studies not measuring HLPSK, so why include these studies ? Again, pg 26-28 are very descriptive and I wonder what point the authors I willing to make. For example, "four papers do not provide a theoretical basis for their use of social capital..." so what does it mean ? I wonder whether the author could streamline the long and boring previous result sections.

The section labelled "themes" is the most interesting compared to the previous ones and, from my perspective, should become the core section of the results. For example, this an Interesting finding about the ambiguous effect of the bonding social capital which might deter HLPSK if abstinence clashes with the norm of the group. But this narrative approach might not require such a huge scoping review, right ? Also, some (valuable) themes are not very much related to social capital, such as "past experiences impacting trust". This example supports my claim at the beginning review: the authors are focusing on relational issues related to HLPSK wider than SC, such as trust. Thus, I wonder whether the results could be reorganised around themes that directly speak to relational aspects of HLPSK, such as bonding, bridging, trust, family, peers, norms,....That would makes much sense given the RQ and would not be a great difficult as the material is there.

The § "what insights applying SNA", pg 32, is coming back to a very boring descriptive style and focused, again on methodological issues. Frankly, this section is not saying much and, sometimes, reads as just a methodology of SNA "Personal network studies collect ego perceptions of alters characteristics and the relationship qualities, which will always be subjective (bottom pg 33) ". This sentences and others do not add to the RQ. Yet, the next § "synthesis of sna studies" is valuable BUT addresses themes that have been already raised (peers, family, ...). For example, on pg 34, there is a nice § about SNA studies and the role of the family members in HLPSK, so I wonder whether having just one section with that theme including both SC studies and SNA studies would make the paper more readable.

**Reviewer**	**2**
**Name**	**Ahlborg, Mikael G.**
**Affiliation**	**Halmstad University, Health and Welfare**
**Date**	**14-Jan-2025**
**COI**	**I have no competing interests.**

Dear Authors,

Thank you for letting me review this interesting paper on help-seeking in marginalized groups and the use of social capital as an explanatory factor for help-seeking behavior. The manuscript has merit, but I feel that there are some points that could benefit the understandability and interpretability of the paper. The introduction is well written and the methodology is sound and follow the common procedures of scoping reviews.

Major concern (related to the entirety of the paper)

This concern is not possible to pin-point to a specific part of the manuscript, which may be troublesome for the authors, however, it is necessary to address. Based on the title and the introduction, I anticipated a focus on socially marginalized groups experiencing discrimination from society based on their social position. This would for example include ethnic minorities, people "outside" of society (based on homelessness, incarceration, long-term unemployment etc). Although this would present a heterogenic group, the group would share a burden of marginalization. Then, by adding mental illness or substance use, you would be able to describe how the concept of social capital could help describe/influence help-seeking in a group experiencing a double burden (Social marginalization + MI/SU).

Reading the method section, search strategy and tables, I was a bit surprised that the marginalized group included people experiencing mental health concerns or substance use (current or former) as an indicator for marginalization. Either the introduction needs a broader definition of marginalized groups, or the inclusion criteria of articles needs to be revised. To help frame the entirety of the manuscript, I would suggest changing the inclusion criteria, but that would require substantial work. However, at this point, I feel that the logic of the results section is rather poor and the understandability questionable. It is well-known that mental illness and substance misuse exists along the social gradient, but in different "forms" and impacting people differently depending on their socioeconomic status. Therefore, the focus of the paper would be clearer if marginalized groups were the base, and mental illness and substance misuse the second layer. Then, you would add social theory and help-seeking to help disentangle the problem with healthcare access you describe initially in your introduction. I believe that the results section could be more processed and well-presented by addressing this issue (more homogenous language that adhere better to each concept for example).

Minor points:

- On page 21, the authors state the following *"In this section, we present the results of the six studies focused on help-seeking".* The following section then presents each article, the method and the results, very thoroughly. I am not familiar with presenting this much "raw data" in scoping reviews. It may be a lack of experience with reading scoping reviews, but I feel that this section adds poorly to the understanding of the results. I would suggest that the authors present this part of the results in a more understandable way, which would include a more processed and reader-friendly presentation, clearly relating to the aim.
- On page 10 the authors state the following "*The growing application of SNA in health research, and its suitability for operationalising and empirically examining* ***social capital concepts****, suggests SNA research may highlight key considerations for understanding help-seeking for MH/SU among people experiencing social marginalization"*. I think I read somewhere else in the text about social capital concepts, in plural. Just to make it perfectly

clear, I think many researchers studying social capital would agree that social capital is one concept, that holds different dimensions and constructs. This may be a misspelling, but if it is not, would need a clarification from the authors.

- In the rationale, the following is stated "*There is limited literature that examines help-seeking as a component of access to MH/SU care, particularly among people experiencing social marginalization"*. I partly agree with this statement. I would say that there is limited literature that examines the **social aspect of** help-seeking intention and behavior… while studies reporting on actual help-seeking for mental health problems have been published over the past decade.
- In Table 2, the term "health-seeking" is used. Should this be changed to help-seeking?
- In the results section, there are statements similar to "none of the six articles were focused on help-seeking". In my opinion, this does not benefit the manuscript. Based on your inclusion criteria, all articles should include a marginalized group, MI/SU, Social capital, and help-seeking in some way. Please be more clear in your language how you have interpreted the extracted information and present what you have found with reference to the areas listed above.
- From my experience, the micro- meso- and macro perspective on social capital is not as common as the individual and network perspective in social capital research. That is, individual social capital is assessed or described as something that is strong or weak based on how it benefits oneself or how an individual interprets the latent resources that may benefit themselves. The network perspective is about what exists between people, that may benefit the group, such as, mutual trust, norms, reciprocity etc. Given that the synthesis on micro- meso- macro is rather short, this may be an explanation. As it stands, it adds little to the results. However, it is not certain that changing to individual and network will yield much more related to the topic, individual social capital is probably overrepresented in the articles. I note that you present "Network culture" later in the synthesis.

As a whole, improving the structure of the results section, by presenting a more processed text would help the manuscript forward.

VERSION 1 - AUTHOR RESPONSE

Reviewer 1 (Lorant)	Reviewer 2 (Ahlborg)	Response
	page 10 "… *examining **social capital concepts**,"* …many researchers agree that social capital is one concept, that holds different dimensions and constructs.<br><br>the rationale, "*There is limited literature that examines help-seeking as a component of access to MH/SU care, particularly among people experiencing social*	We have rephrased as simply 'social capital'.<br><br>We have clarified in the rationale that we mean there is a relatively limited literature for MH/SU help-seeking *for people experiencing social marginalisation*, particularly relational elements.

	marginalization". I partly agree with this statement. I would say that there is limited literature that examines the **social aspect of** help-seeking intention and behavior…	
If the authors are interested into the relational influences of HPSK then why not word the objective with such a wording "relational". Social capital is not the only way to conceptualize and address relationships. Similarly, SNA is more of a perspective then a concept so using SNA in the RQuestion makes the question more fuzzy conceptually. **There is a gap between the motivation of the review and the way the RQ is framed.**		In the introduction section we have added clarification about the motivation for the research, and how we framed of our research questions. We add detail to the reasons for selecting social capital (theory) and social network analysis (analytical approach to studying it) over other theories/methods. We had previously acknowledged the potential for other approaches in our discussion section, but this is now more explicit in the introduction.<br><br>We amended the objective of the review accordingly, using 'relational' terminology as suggested.
Second**, I missed several key papers** that are quite well known in this domain, having used SNA for people with MI. Notice that by definition **people with severe mental illness are quite marginalized and I thus wonder whether adding the criteria of marginalization made sense at all**.	The focus of the paper would be clearer if marginalized groups were the base, and mental illness and substance misuse the second layer.	The reviewers held opposing views about the inclusion criteria. Lorant arguing that marginalisation beyond experiencing mental ill-health (and presumably substance use) seemed superfluous given the marginalisation experienced by people with serious mental illnesses in itself. In contrast, Ahlborg argues for marginalisation experiences *other* than MH/SU to be the focus, and (as we interpret their comments) only to include studies where there is marginalisation beyond MH/SU.<br><br>Our population of concern are people experiencing social marginalisation. Our focus on marginalised groups is because relational influences on people experiencing social marginalisation likely differ from more common research samples (e.g., general

		population/students/service users).<br><br>Due to a very limited literature identified when adopting the approach advocated by Ahlborg (n=5), we included studies where the marginalisation experience was mental ill-health or substance use (as advocated by Lorant). Although participants in the latter studies may not have *previously* been socially marginalised, living with mental illness or substance use exposes people to stigma and exclusion. These papers add depth and nuance to our review. We have added clarification of this in the methods section.<br><br>We tightened our criteria in relation to MH/SU, to increase the likelihood that these were marginalised participants, and only retained papers where the population had *pre-existing* MH/SU problems to the level of service receipt or diagnosis that would indicate exposure to marginalisation. This resulted in the exclusion of three papers (Raymond 2009, Takehashi et al 2009, Wester et al 2015).<br><br>As a scoping review, we did not want to include every possible paper describing help-seeking among people with different marginalised statuses, but to identify evidence gaps and broad characterisation of the evidence base. We have added clarity within methods about the reasons for our selection - being based on common experiences of social disadvantage in the UK.<br><br>The papers provided by Lorant were identified in our searches and excluded at title and abstract stage because they do not describe *help-*

		seeking within title and abstract. We have re-reviewed these at full text, and are satisfied these do not discuss help-seeking and therefore do not meet our criteria. We thank Lorant for highlighting these papers, which will be useful in framing our future work in relation to psychiatric service use.
Third, social network analysis and social capital are eligibility criteria but these terms are not on the same level: social capital is a concept where SNA is a perspective or a group of methods. So they don't align.		We investigated theoretical (SC) and methodological (SNA) applications for studying the relational influences on help-seeking. We did not intend these to align, and have made clarifications through out the manuscript to explain this more clearly.
	In Table 2, the term "health-seeking" is used. Should this be changed to help-seeking?	Correction made.
Results<br>From the middle of page 15 to page 21 we are given a long description of the study features, but this is a long and somewhat boring section that do not provide much information regarding the RQ. For example almost one page is devoted to describe the timing and location (including a map!!) of the studies and the information given in the figures duplicate somewhat the text. Is all this much necessary ? In a way, the interesting stuff begins on page 21. So I would strongly suggest to downsize this first descriptive section.<br><br>Theoretical orientations- on page 26, "in addition to Perry and Pescosolido 2015", this seems to including studies not	As a whole, improving the structure of the results section, by presenting a more processed text would help the manuscript forward<br><br>On page 21, the authors state the following *"In this section, we present the results of the six studies focused on help-seeking"*. The following section then presents each article, the method and the results, very thoroughly. I am not familiar with presenting this much "raw data" in scoping reviews. It may be a lack of experience with reading scoping reviews, but I feel that this section adds poorly to the understanding of the results. I would suggest that the authors present this part of the results in a more understandable way, which would include a	To improve readability and precision, we have substantially reviewed the results section by:<br>- Reducing the amount of descriptive information and the results of individual studies, instead referring the reader to the data extraction tables.<br>- Providing short sections about SC and SNA<br>- Integrating the thematic findings about relational influences on help-seeking<br>- Providing data tables in the main manuscript for all studies included in the review

measuring HLPSK, so why include these studies ? Again, pg 26-28 are very descriptive and I wonder what point the authors I willing to make. For example, "four papers do not provide a theoretical basis for their use of social capital..." so what does it mean? I wonder whether the author could streamline the long and boring previous result sections	more processed and reader-friendly presentation, clearly relating to the aim.<br><br>there are statements similar to "none of the six articles were focused on help-seeking". In my opinion, this does not benefit the manuscript. Based on your inclusion criteria, all articles should include a marginalized group, MI/SU, Social capital, and help-seeking in some way. Please be more clear in your language how you have interpreted the extracted information	
The section labelled "themes" is the most interesting compared to the previous ones and, from my perspective, should become the core section of the results. For example, this an interesting finding about the ambiguous effect of the bonding social capital which might deter HLPSK if abstinence clashes with the norm of the group. But this narrative approach might not require such a huge scoping review, right? Also, some (valuable) themes are not very much related to social capital, such as "past experiences impacting trust". This example supports my claim at the beginning review: the authors are focusing on relational issues related to HLPSK wider than SC, such as		We have restructured our results section as above to reflect these suggestions.<br><br>We have provided greater detail in the results section about how papers used trust in relation to social capital theory. We clarify where trust is described as network level attribute/resource, a characteristic of ego/alter or a relationship quality. We highlight the disagreement about the position of trust in relation to social capital in our discussion section.

trust. Thus, I wonder whether the results could be reorganised around themes that directly speak to relational aspects of HLPSK, such as bonding, bridging, trust, family, peers, norms,....That would makes much sense given the RQ and would not be a great difficult as the material is there.		
The "what insights applying SNA", pg 32, is coming back to a very boring descriptive style and focused, again on methodological issues. Frankly, this section is not saying much and, sometimes, reads as just a methodology of SNA "Personal network studies collect ego perceptions of alters characteristics and the relationship qualities, which will always be subjective (bottom pg 33) ". This sentences and others do not add to the RQ. Yet, the next "synthesis of sna studies" is valuable BUT addresses themes that have been already raised (peers, family, ...). For example, on pg 34, there is a nice § about SNA studies and the role of the family members in HLPSK, so I wonder whether having just one section with that theme including both SC studies and SNA studies would make the paper more readable.		As above – we have revised the presentation of results to improve this.<br><br>We retained the findings about the authors reflections on the methodological utility of SNA in line with our question about the insights (including methodological) from the application of SNA. However, we have amended this to be less descriptive.
	From my experience, the micro- meso- and macro perspective on social capital is not as	We understand micro as ‘individual level’, meso as ‘network level’, and macro as ‘societal level’ and so our presentation can be interpreted as consistent with Ahlborg’s

	common as the individual and network perspective in social capital research. That is, individual social capital is assessed or described as something that is strong or weak based on how it benefits oneself or how an individual interprets the latent resources that may benefit themselves. The network perspective is about what exists between people, that may benefit the group, such as, mutual trust, norms, reciprocity etc. Given that the synthesis on micro meso-<br>macro is rather short, this may be an explanation. As it stands, it adds little to the<br>results. However, it is not certain that changing to individual and network will yield much more related to the topic, individual social capital is probably overrepresented in the articles.<br>I note that you present "Network culture" later in the synthesis.	description, and we have clarified this within the manuscript.<br><br>We have expanded our discussion of the limited literature covering network and societal level influences, and highlight that network culture remain underexplored.

VERSION 2 - REVIEW

**Reviewer**	**2**
**Name**	**Ahlborg, Mikael G.**
**Affiliation**	**Halmstad University, Health and Welfare**
**Date**	**27-Mar-2025**
**COI**	

Dear authors,

I appreciate the major revisions made to the manuscript. However, my concern about the focus of the study and the broad target group remains and needs to be further explained. I absolutely agree with reviewer 1 that there exists social marginalization and stigmatization for people experiencing mental illness. The issue I am trying to raise is that social marginalization itself is a broad term that includes different types of marginalization. See this paper for further information: Fluit, S., Cortés-García, L. & von Soest, T. Social marginalization: A scoping review of 50 years of research. Humanit Soc Sci Commun 11, 1665 (2024). https://doi.org/10.1057/s41599-024-04210-y.

Some of these types of social marginalization impose a burden on individuals from birth, unlike others, which means that being socially marginalized related to culture or ethnicity while experiencing mental health problems/mental illness arguably is different than the social marginalization or stigmatization of mental illness within a cultural/ethnical majority in a community/country. I therefore ask the authors to either provide a convincing argument to the included aspects of social marginalization or to remove it and focus on people experiencing mental health problems/mental illness as a large and disadvantaged group.

Although a scoping review is differential to a systematic review, in my experience it is more common to include papers that extend beyond a defined concept of interest, to include related papers where similar terms have been used, rather than limiting the inclusion criteria and exclude papers within concepts of interest. I understand that papers on social marginalization related to help-seeking and social capital OR SNA may be limited, but at this point I feel that the methodological approach to inclusion/exclusion is insufficient to make an argument that the research field has been scoped. Especially concerning cultural and ethnic/racial aspects of social marginalization.

Additionally, I feel that the change from mental illness or mental ill-health to the term mental health is a bit confusing since people are not treated for “mental health”. I fail to see how this change adds clarity to the paper.

**Reviewer**	**3**
**Name**	**Chantry, Megane**
**Affiliation**	**UCLouvain, IRSS**
**Date**	**14-Mar-2025**
**COI**	**None**

Generally speaking, the paper has been reworked, but the initial theoretical and methodological orientations have remained the same.

1. The objective of the review is clear; however I am not convinced that the theory of social capital and the method of SNA should orient the research question. To me, it is rather "obvious" that studying the influence of social relationships on help-seeking behaviour will discuss the theory of social capital (even if they are other theories). In my opinion, this could be rather an element of the discussion rather than a inclusion criteria. With the explananations in the manuscrpit, I understand why they focus on Social capital theory and SNA, but I am not convinced that it should orient the research question.

2. The review focuses on help-seeking (RQ), but I think there is a lack of coherence regarding the definition of help-seeking as some results are broader and also discuss service access: "Service providers were sometimes the only sources of bridging and linking capital in peoples' lives and vital for accessing support and sustaining engagement with it". The definition of help seeking should be clarified: it is defined as a component of accessing treatment or support and as "actively seeking assistance, support or guidance, including professional services". Therefore, it seems to me that help-seeking is not restricted to service use but could involve service use/access.

3. In line with the issue of definition explained above, I think that the review should include papers on the social networks of people with mental illness using psychiatric services as they are people who are seeking help for MH problems.

4. The use of the terms "mental health problems", "mental ill-health", "substance use", "substance use problems" or "substance problems use" should be more consistent throughout the article, as should the use of abbreviation MH/SU.

5. Finally, the paper should be revised in terms of written English, as a few errors were detected when reading the article.

VERSION 2 - AUTHOR RESPONSE

Reviewer 3 questioned whether social capital and SNA should orient the research question. They additionally comment that the objective is clear and that they understand why the focus is on social capital and social network analysis.<br><br>Reviewer 3 felt it is "obvious" that studying the influence of social relationships on help-seeking behaviour will discuss the theory of social capital (even if they are other theories) and that this may	We thank reviewer 3 for noting the clarity of our objective. We agree the focus of the paper should be understanding relational influences on help-seeking. Our objectives and eligibility criteria have been reframed to bring help-seeking to the forefront.<br><br>The discussion of social capital in the introduction section more strongly differentiates between social capital focused approaches to understanding relational effects, and other individual and structural approaches. We have explained more clearly in the methods section why we focused on social capital and SNA as search terms when identifying papers. There is discussion of the implications of this in the limitations section at the end of the document.

be better within the discussion rather than an inclusion criteria.	Despite it seeming obvious that social capital would be discussed when studying relational influences on help-seeking, the results of our review highlight that this is not the case. As such we maintain that this review highlights important findings.
Reviewer 3 requested clarification of the definition of help-seeking, particularly around its relationship to service use.	We have added a section to the introduction which clarifies how we have interpreted help-seeking (i.e., the act of seeking help or additional help, not whether or not that act results in service use). We have stated we are not interested in the quality of help provided, but understanding how social relations can influence people's decision-making and choices to act to seek help. We have added clarity in the eligibility criteria section of the methods to explain what we considered as help-seeking.
Reviewer 3 questioned whether our review should include papers about the social networks of people using psychiatric services	Many of these papers were identified during our database searches. We rejected papers which either focused solely on people using services (as opposed to engaging with the transitionary processes of deciding to seek help), or which looked at the general population rather than focusing on the intersectional issues around what we regarded as social marginalisation. Papers which met our eligibility criteria were included. We acknowledge there are papers which explore the networks of people who are *receiving* help, but have excluded them from our study as we are focused on people *seeking* help.
Reviewer 2 commented that our conceptualisation of social marginalisation was too narrow.	We agree with the point about lifelong marginalisation due to socio-demographic characteristics may differentially influence help-seeking compared to the marginalisation experiences we included. As the reviewer points out in their response and the provided paper, marginalisation does not have a consensus definition or what is included or not.<br><br>We have made it clear throughout the paper we are not focusing on socio-demographic factors, such as gender and ethnicity (not to mention disability, sexual orientation, age and many other factors). Our focus is on marginalisation experiences associated with multiple disadvantage: criminal justice involvement, homelessness, substance use, addiction and mental ill-health. Our central interest is around people who might seek help for mental health or substance use issues but who experience social marginalisation for reasons beyond their socio-demographic characteristics. As such, as the reviewer requests, this included people with pre-existing mental illnesses.
Reviewer 2 felt we could have been wider in our search criteria, taking a broader viewer. Our	As a scoping review, we defined the inclusion and exclusion criteria, as set out in the manuscript, that enabled us to answer the research questions. Our

focus might have excluded some cultural, ethnic and racial aspects of marginalisation.	scoping review followed an established rigorous methodology. We did include papers identified outside of our search which met the eligibility criteria, however the nature of setting these criteria means that other things are out of scope. We have described how we viewed the three aspects of our inclusion/exclusion criteria in the eligibility criteria section, which now has a paragraph clearly demonstrating our thinking on each.<br><br>We agree that our focus on marginalisation excluded papers that focused on help-seeking for MH/SU among people from ethnic and cultural minorities without additional marginalisation due to homelessness, justice involvement, mental ill-health or substance use problems. This was not the question we sought to answer. We have added reflection on this to the limitations section of our study.
Reviewers 2 and 3 both commented on the consistency of terms (particularly around describing mental health) and other writing style.	We have gone through the paper and been more consistent in our language. We have also given the paper an additional close proofreading and editing of phrasing.
